# Deciphering the prognostic significance of anoikis-related lncRNAs in invasive breast cancer: from comprehensive bioinformatics analysis to functional experimental validation

**DOI:** 10.18632/aging.205376

**Published:** 2024-01-05

**Authors:** Wenge Dong, Jiejing Li, Zhigang Zhuang

**Affiliations:** 1 Department of Breast Surgery, Shanghai Key Laboratory of Maternal Fetal Medicine, Shanghai Institute of Maternal-Fetal Medicine and Gynecologic Oncology, Shanghai First Maternity and Infant Hospital, School of Medicine, Tongji University, Shanghai 200092, China

**Keywords:** breast cancer, anoikis, long non-coding RNA, prognostic biomarker, immune microenvironment characteristics

## Abstract

The global prevalence of breast cancer necessitates the development of innovative prognostic markers and therapeutic strategies. This study investigated the prognostic implications of anoikis-related long non-coding RNAs (ARLs) in invasive breast cancer (IBC), which is an area that has not been extensively explored. By integrating the RNA sequence transcriptome and clinical data from The Cancer Genome Atlas (TCGA) database and employing advanced regression analyses, we devised a novel prognostic model based on ARL scores. ARL scores correlated with diverse clinicopathological parameters, cellular pathways, distinct mutation patterns, and immune responses, thereby affecting both immune cell infiltration and anticipated responses to chemotherapy and immunotherapy. Additionally, the overexpression of a specific lncRNA, *AL133467.1*, significantly impeded the proliferation and migration, as well as possibly the anoikis resistance of breast cancer cells. These findings highlight the potential of the ARL signature as a robust prognostic tool and a promising basis for personalized IBC treatment strategies.

## INTRODUCTION

Breast cancer poses a significant global health challenge [1] and continues to be one of the most prevalent cancers worldwide, with 2.26 million reported cases in 2020. In addition, it is the main factor affecting female cancer-related fatalities [2]. Invasive breast cancer (IBC) is the most prevalent type of breast cancer-related mortality. Although the prognosis has improved for most patients with IBC, inter-individual heterogeneity can still result in a poor prognosis for some patients [3]. Therefore, it is imperative to identify novel biomarkers as soon as possible to improve IBC patient diagnosis and therapy.

When cells separate from the extracellular matrix, a type of programmed cell death known as anoikis occurs. [4]. In normal cells, this process functions as a protective mechanism that prevents the survival and proliferation of cells detached from their normal tissue architecture. However, in cancer cells, resistance to anoikis is a crucial step in acquiring metastatic potential [5]. Cancer cells typically separate from the original tumor and infiltrate the neighboring tissues as the tumor grows. These cells must resist anoikis and survive in the absence of attachment to the extracellular matrix [4]. Anoikis resistance can be mediated by various mechanisms, including alterations in cell-surface receptor expression, activation of survival pathways, and changes in the expression of pro- and anti-apoptotic proteins [6]. According to current findings, anoikis is critical for the development of gastric cancer [7], esophageal squamous cell carcinoma [8], and breast cancer [9].

Long noncoding RNAs (lncRNAs) are involved in various biological activities and play crucial roles in the regulation of gene expression [10]. Dysregulated expression of lncRNAs has been observed in cancer, where they function as oncogenes or tumor suppressors [11]. The mechanisms by which lncRNAs regulate cancer development are diverse and complex and involve interactions with DNA, RNA, and proteins [12]. An enhanced understanding of lncRNA functions in tumor biology may pave the way for innovative cancer therapies. However, the precise roles of anoikis-related lncRNAs (ARLs) in invasive breast cancer have not yet been fully elucidated.

In the present study, we systematically explored the role of ARLs in IBC. We identified IBC-related ARLs and created a prognostic model that can direct prognostic predictions and clinical treatment decisions. Furthermore, we thoroughly examined a key ARL prognostic gene, *AL133467.1*, to validate its association with migration, proliferative capacity, and anoikis in breast cancer cells. By uncovering the molecular mechanisms underlying IBC progression, our findings offer valuable insights and present a potential novel strategy for the diagnosis and treatment of this disease.

## RESULTS

### Identification of anoikis-related differentially expressed LncRNAs

**Figure 1:**
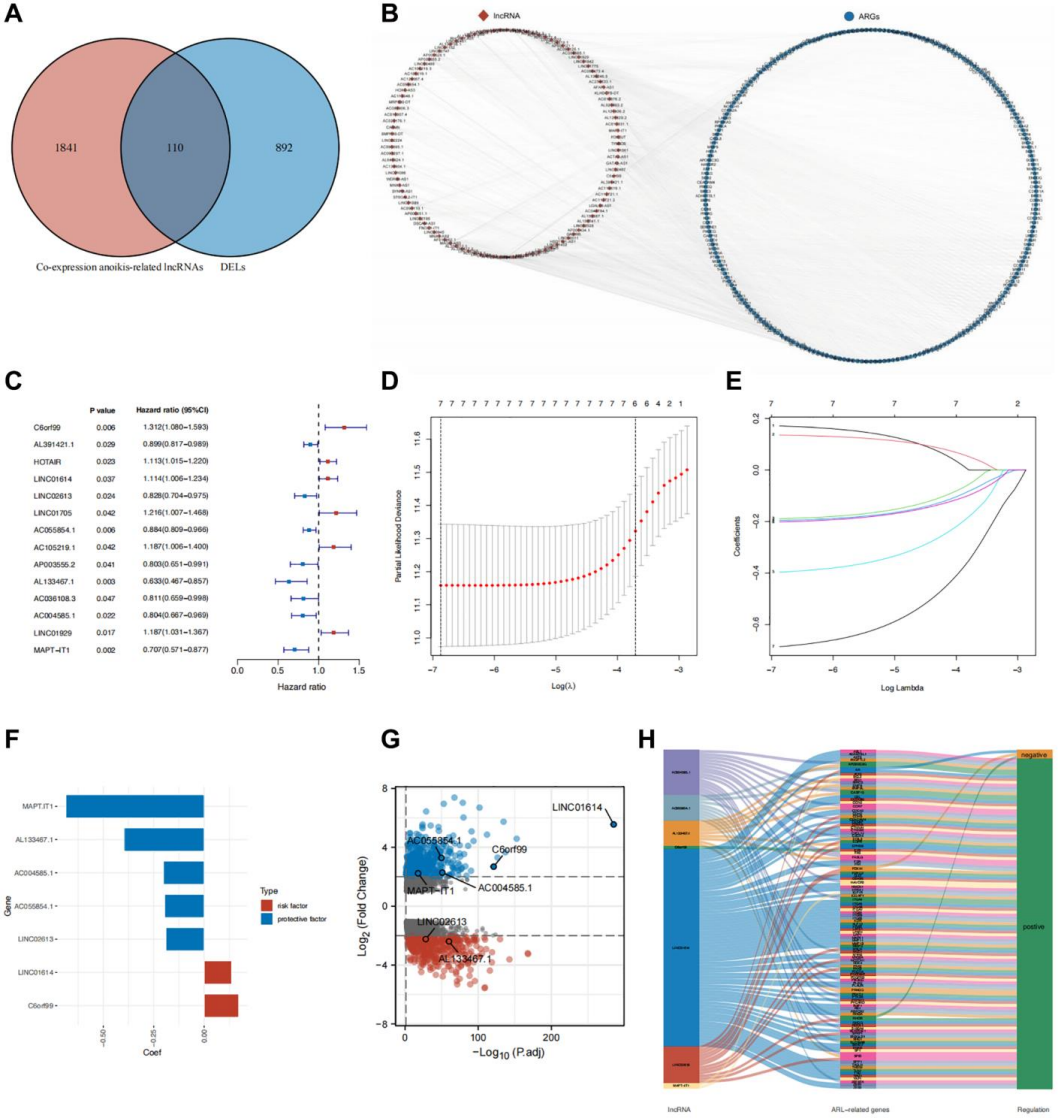
**Identification of core DELs related to anoikis and construction of a prognostic model.** (**A**) Venn diagram illustrating the screening process for DELs related to anoikis. (**B**) Network diagram depicting the relationship between the identified anoikis-related DELs and their corresponding genes. (**C**) Results of univariate Cox analysis. (**D**, **E**) Results of LASSO regression analysis (**F**) Coefficients of the seven core lncRNAs based on LASSO analysis. (**G**) Volcano plot displaying the differential expression of the seven core lncRNAs in normal and tumor tissues. (**H**) Sankey diagram illustrating the correlation between anoikis-related DELs and genes in breast cancer.

We identified 1002 differentially expressed lncRNAs (DELs) between normal breast and IBC samples. In a previous study, 434 anoikis-related genes were identified [13]. We then performed co-expression analysis to identify 1951 lncRNAs that are co-expressed with anoikis-related genes. The 1002 DELs intersected with the 1951 co-expressed lncRNAs, resulting in the selection of 110 anoikis-related DELs (Figure 1A). The co-expression network of anoikis-related DELs and genes is shown in Figure 1B.

### Screening prognostic anoikis-related DELs and construction of a prognostic model

We identified 14 lncRNAs (C6orf99, AL391421.1, HOTAIR, LINC01614, LINC02613, LINC01705, AC055854.1, AC105219.1, AP003555.2, AL133467.1, AC036108.3, AC004585.1, LINC01929, and MAPT-IT1) as prognostic genes (*P* < 0.05) through univariate Cox analysis of 110 anoikis-related DELs (Figure 1C). We subsequently screened seven core prognostic genes (C6orf99, LINC01614, LINC02613, AC055854.1, AL133467.1, AC004585.1, and MAPT-IT1; *P* < 0.05) by Kaplan-Meier survival analysis (Supplementary Figure 1). The breast cancer patients in TCGA were randomly distributed into test and training cohorts. Table 1 illustrates that the baseline traits of patients with breast cancer did not vary significantly between the training and test sets. For the training set, the seven prognostic lncRNAs were integrated into Least Absolute Shrinkage and Selection Operator (LASSO) regression analysis to establish a prognostic model (Figure 1D, 1E). This model was constructed using the following formula for the anoikis-related lncRNA (ARL) score: ARL score = C6orf99 × 0.171007158825575 + LINC01614 × 0.135601559840202 + LINC02613 × −0.189357717333547 + AC055854.1 × −0.195901072958402 + AL133467.1 × −0.396564264243533 + AC004585.1 × −0.201708797422736 + MAPT.IT1 × −0.685621403207209 (Figure 1F). As shown in Figure 1G, C6orf99, LINC01614, AC055854.1, AC004585.1, and MAPT.IT1 were highly expressed in breast cancer, whereas LINC02613 and AL133467.1 were expressed at lower levels. Additionally, Figure 1H shows that the seven core prognostic lncRNAs positively correlated with most of the corresponding co-expressed anoikis-related genes.

**Table 1:** Comparison of clinical feature between train and test sets.

**Clinical feature**		**Total (*N* = 1065)**	**Test (*N* = 532)**	**Train (*N* = 533)**	***P* value**
Age	–	–	59 (48, 68.25)	57 (48, 65)	0.0543
T	T1&T2	893 (83.85%)	442 (83.08%)	451 (84.62%)	0.5961
	T3&T4	169 (15.87%)	88 (16.54%)	81 (15.2%)	
	Unknown	3 (0.28%)	2 (0.38%)	1 (0.19%)	
N	N0	494 (46.38%)	243 (45.68%)	251 (47.09%)	0.8649
	N1&N2&N3	551 (51.74%)	275 (51.69%)	276 (51.78%)	
	Unknown	20 (1.88%)	14 (2.63%)	6 (1.13%)	
M	M0	874 (82.07%)	441 (82.89%)	433 (81.24%)	0.1835
	M1	21 (1.97%)	7 (1.32%)	14 (2.63%)	
	Unknown	170 (15.96%)	84 (15.79%)	86 (16.14%)	
Pathologic stage	I&II	784 (73.62%)	394 (74.06%)	390 (73.17%)	0.8295
	III&IV	258 (24.23%)	127 (23.87%)	131 (24.58%)	
	Unknown	23 (2.16%)	11 (2.07%)	12 (2.25%)	
ER	Negative	229 (21.5%)	111 (20.86%)	118 (22.14%)	0.6049
	Positive	793 (74.46%)	402 (75.56%)	391 (73.36%)	
	Unknown	43 (4.04%)	19 (3.57%)	24 (4.5%)	
PR	Negative	328 (30.8%)	159 (29.89%)	169 (31.71%)	0.518
	Positive	692 (64.98%)	352 (66.17%)	340 (63.79%)	
	Unknown	45 (4.23%)	21 (3.95%)	24 (4.5%)	
HER2	Negative	545 (51.17%)	274 (51.5%)	271 (50.84%)	0.7997
	Positive	154 (14.46%)	75 (14.1%)	79 (14.82%)	
	Unknown	366 (34.37%)	183 (34.4%)	183 (34.33%)	

### Application and validation of the prognostic model

**Figure 2:**
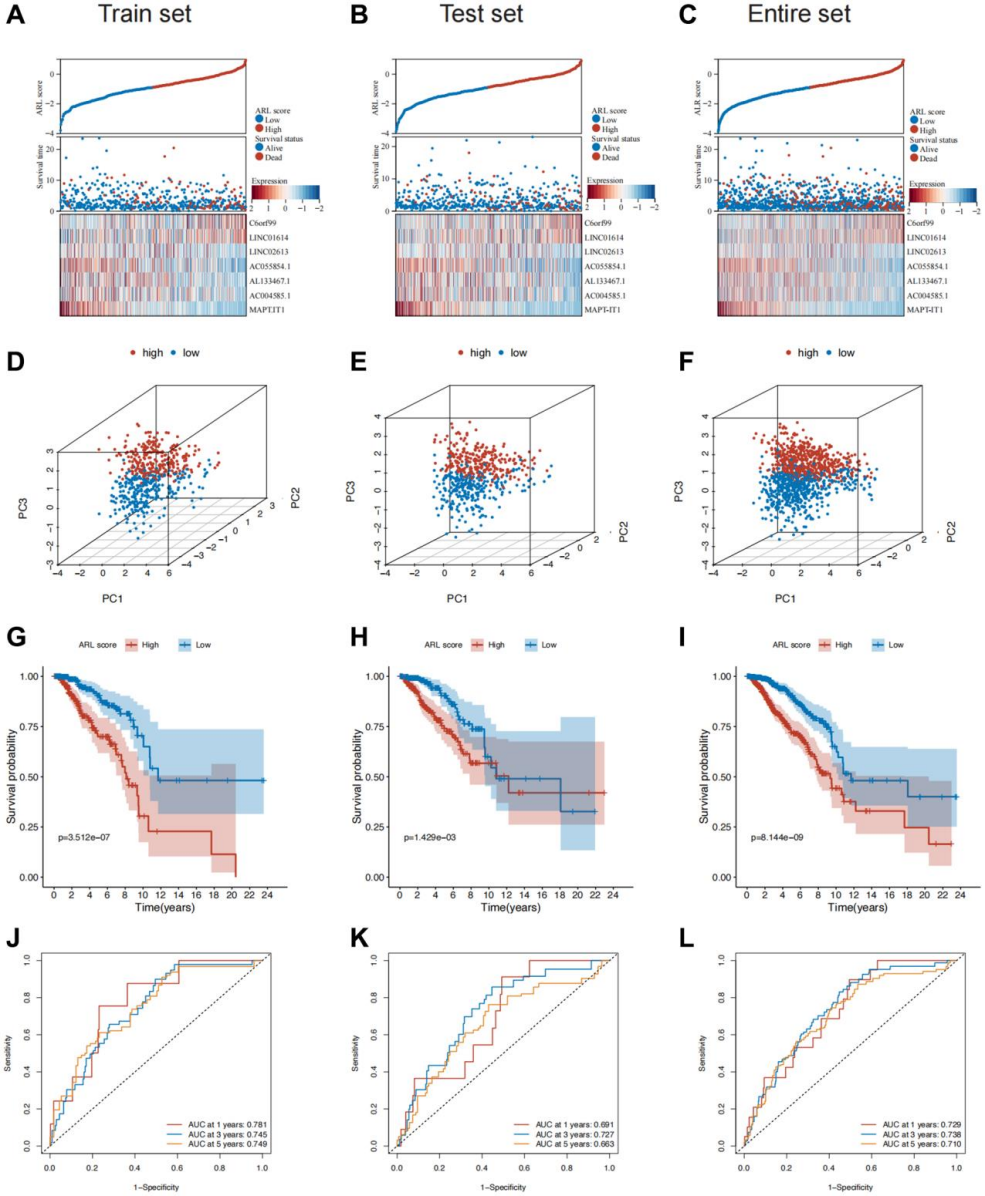
**Construction and validation of the prognostic model in the train, test, and entire sets.** (**A**–**C**) Distribution of ARL scores, survival status, and expression levels of the seven core lncRNAs in the train, test, and entire sets. (**D**–**F**) Results of principal component analysis (PCA) in the train, test, and entire sets. (**G**–**I**) Survival differences between the low and high ARL score groups in the train, test, and entire sets. (**J**–**L**) Results of receiver operating characteristic (ROC) analysis of the prognostic model in the train, test, and entire sets.

Patients in both sets were further classified into high and low ARL score groups based on the median ARL score. The ARL score distribution, survival status, and expression levels of the seven core lncRNAs in the low- and high ARL score groups are shown in Figure 2A–2C. These results illustrate the intuitive differences between the two groups in the training, testing, and entire sets, which were supported by Principal Component Analysis (PCA) (Figure 2D–2F).

Moreover, the low ARL score group exhibited significantly better overall survival (OS) compared with the high ARL score group (Figure 2G–2I). This trend was also observed for other clinical characteristics such as TNM stage, grade, and age (Supplementary Figure 2A). The 1-, 3-, and 5-year area under the curve (AUC) values of the prognostic model were 0.729, 0.738, and 0.710, respectively (Figure 2J–2L). In addition, univariate Cox regression analysis showed that the N stage, M stage, pathological stage, and ARL score were predictors of survival in the training set, test set, and entire set, whereas multivariate analysis indicated that the ARL score was the only common independent predictive factor for breast cancer patients across all sets (Supplementary Figure 3). These findings suggest that the model has a relatively good predictive ability for prognosis.

### Correlation between clinical characteristics and the prognostic model

**Figure 3:**
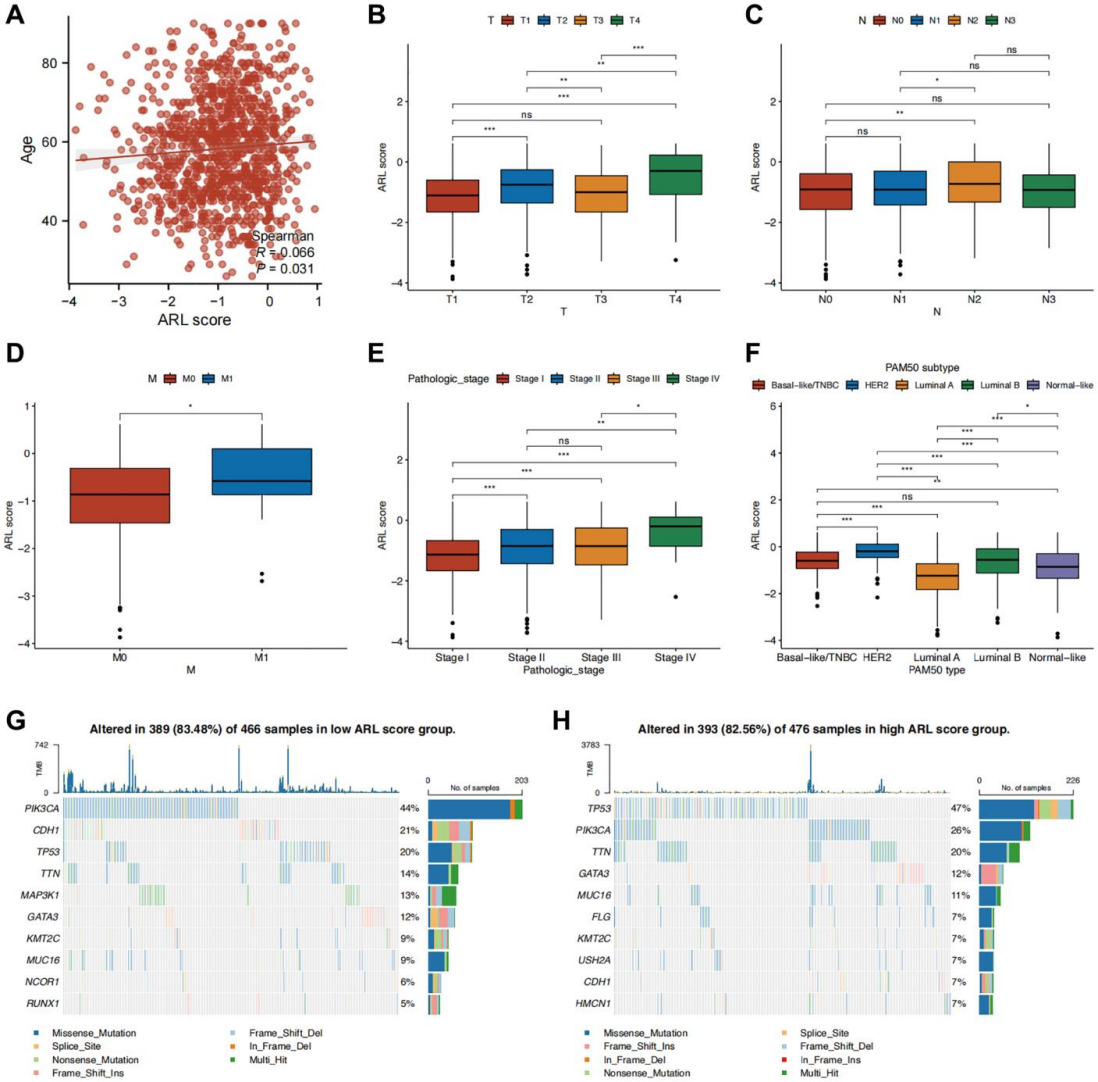
**Correlation between clinical characteristics and the prognostic model.** (**A**–**F**) Clinical characteristics differences between the high and low ARL score groups. (**G**, **H**) Waterfall plots displaying the mutation frequencies of the top 10 genes in the high and low ARL score groups. Statistical significance symbols: ns, *p* ≥ 0.05; ^*^p < 0.05; ^**^p < 0.01; ^***^p < 0.001.

Additionally, we investigated the clinical significance of a prognostic model utilizing ARLs by assessing their association with clinical features. As shown in Figure 3A, a significant correlation was observed between age and the ARL score (*P* < 0.05). Individuals diagnosed with T4, N2, M1, and stage IV conditions demonstrated the most elevated ARL scores, signifying that elevated ARL scores are associated with advanced TNM and pathological stages. (Figure 3B–3E) (*P* < 0.05). Among the PAM50 breast cancer subtypes, patients with HER2-enriched breast cancer had the highest ARL scores (*P* < 0.001), whereas those with luminal A breast cancer had the lowest scores (Figure 3F) (*P* < 0.05). Interestingly, the frequency of PIK3CA gene mutation was highest (44%) in the group with a low ARL score, in contrast to a mutation rate of 26% in the group with a high ARL score. In the high ARL score group, *TP53* emerged as the gene with the highest occurrence of mutations, with a frequency of 47%, whereas the low ARL score group had a mutation rate of only 20% (Figure 3G, 3H). Furthermore, to confirm that the ARL score could differentiate patients with varying prognoses across different breast cancer subtypes, we conducted survival analyses stratified by the ARL score within each breast cancer subtype. The results of our study suggest that patients with low ARL scores consistently had better prognoses (*P* < 0.05) in all four breast cancer subtypes, namely, basal-like/TNBC, luminal A, luminal B, and normal-like. Although a comparable pattern was observed in patients with HER2-amplified breast cancer, the difference was not statistically significant (*P* > 0.05), possibly because of the small number of participants in this specific patient cohort (Supplementary Figure 2B). These findings suggest the potential utility of the ARL-based prognostic model in aiding the diagnosis, prognostication, and formulation of treatment strategies.

### Developing the nomogram to predict the outcome of IBC

**Figure 4:**
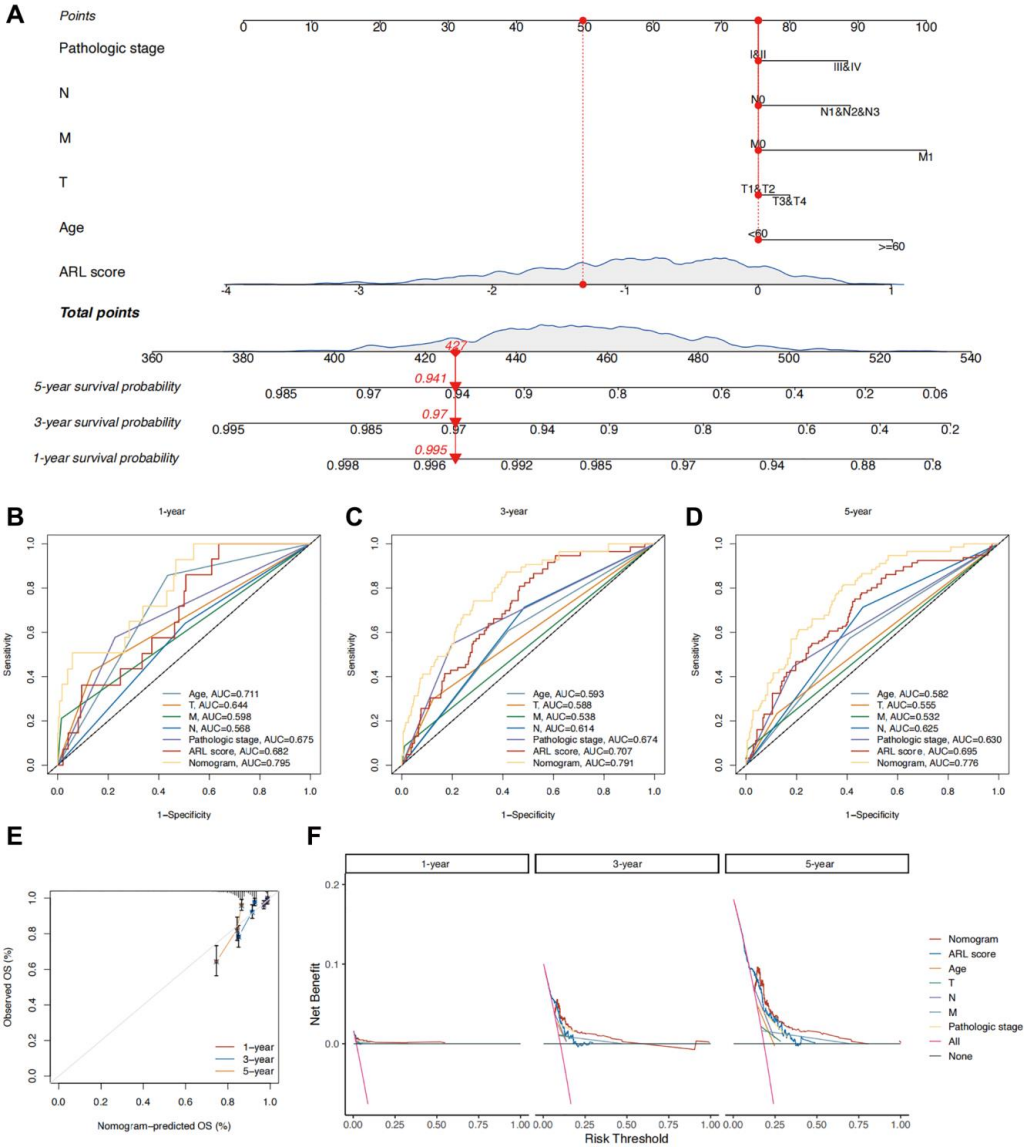
**Construction of a nomogram for predicting the prognosis of IBC patients.** (**A**) Nomogram predicting 1-, 3-, and 5-year overall survival (OS) of IBC patients. (**B**–**D**) Time-independent ROC curves comparing the predictive performance of the nomogram with other prognostic indicators. (**E**) Calibration plots demonstrating the predictive accuracy of the nomogram. (**F**) Decision curve analysis (DCA) assessing the clinical utility of the nomogram.

To improve the practicality of the prognostic model, the ARL scores were integrated with prognostic clinical characteristics to develop a nomogram that predicts the overall survival (OS) of patients with IBC (Figure 4A). To assess the superiority of the nomogram in predicting outcomes, various parameters, such as age, T stage, N stage, M stage, pathological stage, and ARL score, were evaluated using a Receiver Operating Characteristic (ROC) curve. As shown in Figure 4B–4D, the AUC values for predicting the 1-, 3-, and 5-year outcomes using the nomogram (0.795, 0.791, and 0.776, respectively) were higher compared to age (0.711, 0.593, and 0.582, respectively), T stage (0.644, 0.588, and 0.555, respectively), N stage (0.568, 0.614, and 0.625, respectively), M stage (0.598, 0.538, and 0.532, respectively), pathological stage (0.675, 0.674, and 0.630, respectively), and ARL scores (0.682, 0.707, and 0.695, respectively). This indicates the strong ability of the nomogram to predict the prognosis of IBC. Figure 4E shows a calibration plot, indicating that the nomogram exhibited a favorable capacity for predicting patient prognosis. Based on the decision curve analysis (DCA) results for 1-, 3-, and 5-year periods, it can be inferred from Figure 4F that the nomogram exhibited superior clinical usefulness compared to alternative factors. The nomogram prediction model, which is based on ARL scores and clinicopathological features, has been thoroughly validated using various approaches and has demonstrated robust predictive capacity and clinical usefulness.

### Function enrichment analysis

**Figure 5:**
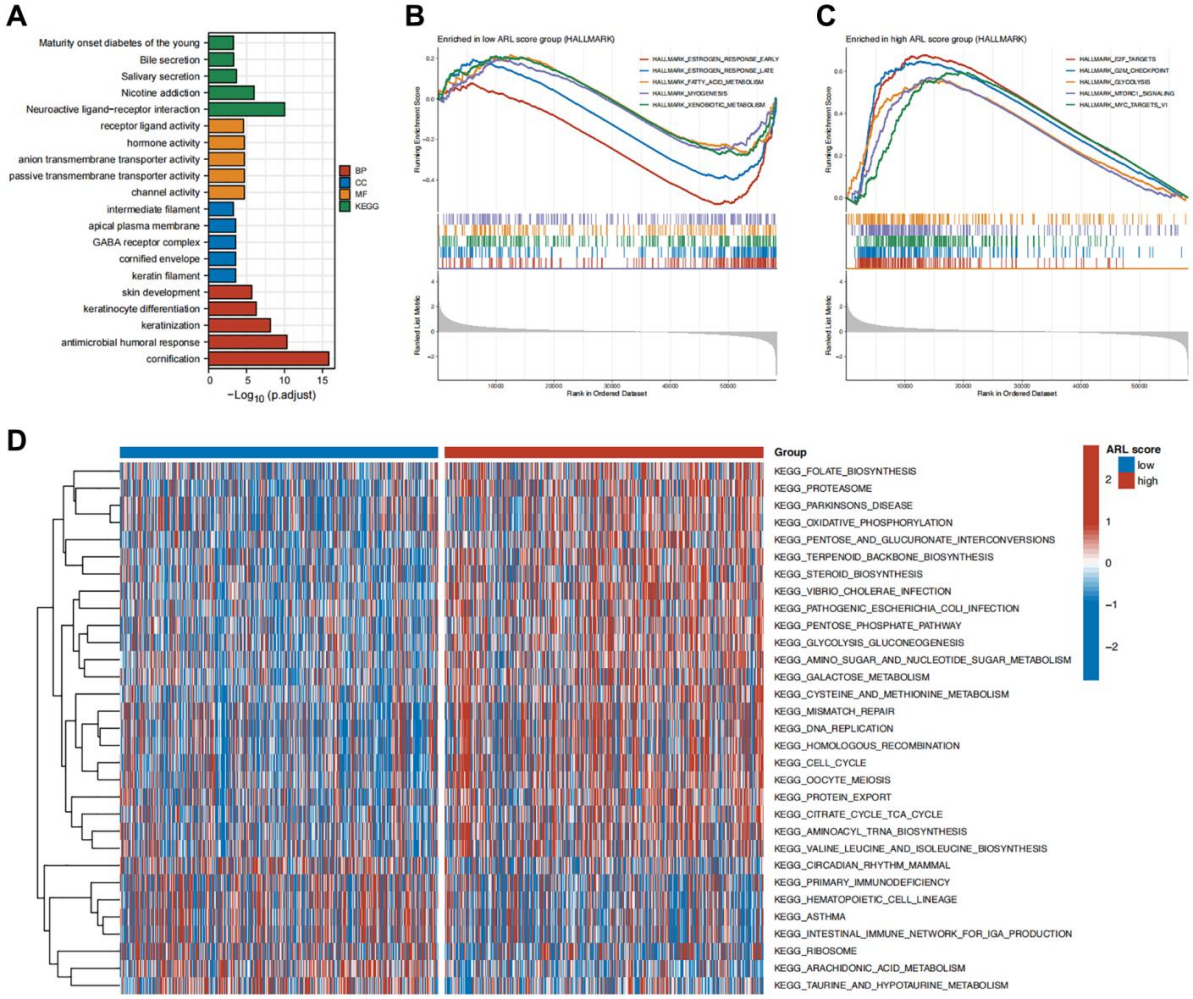
**Functional enrichment analysis.** (**A**) Enrichment results of Gene Ontology (GO) and Kyoto Encyclopedia of Genes and Genomes (KEGG) for the differentially expressed genes (DEGs) between high versus low ARL score groups. (**B**) Top five pathways enriched in the low ARL score group. (**C**) Top five pathways enriched in the high ARL score group. (**D**) Differential pathways between high versus low ARL score groups.

To clarify the possible biological roles and pathways linked to the ARL signature, we performed various enrichment analyses such as GSEA, GSVA, GO, and KEGG. The results of the GO and KEGG enrichment analyses in Figure 5A show a significant correlation between differentially expressed genes (DEGs) in the low and high ARL score groups and signal transduction-related biological functions, including channel activity, passive transmembrane transporter activity, anion transmembrane transporter activity, hormone activity, and receptor ligand activity.

Additionally, we conducted GSEA enrichment analysis to compare the groups with low and high ARL scores and identify distinct biological functions and pathways between the two groups. Figure 5B, 5C illustrate that the group with low ARL scores had a notable enhancement in biological processes, including early estrogen response, late estrogen response, fatty acid metabolism, muscle formation, and xenobiotic metabolism. In contrast, the group with high ARL scores exhibited notable enrichment in pathways including E2F targets, G2/M checkpoint, glycolysis, MTORC1 signaling, and MYC targets.

Furthermore, GSVA revealed 31 distinct pathways that differed between IBC patients with low and high ARL scores. Patients with high ARL scores exhibited positive associations with pathways such as mismatch repair, DNA replication, homologous recombination, and the cell cycle, as shown in Figure 5D, whereas the low ARL score group had negative associations. These results indicated that these physiological processes may be linked to poor outcomes in patients with IBC.

### Immune infiltration characteristics of the TME

**Figure 6:**
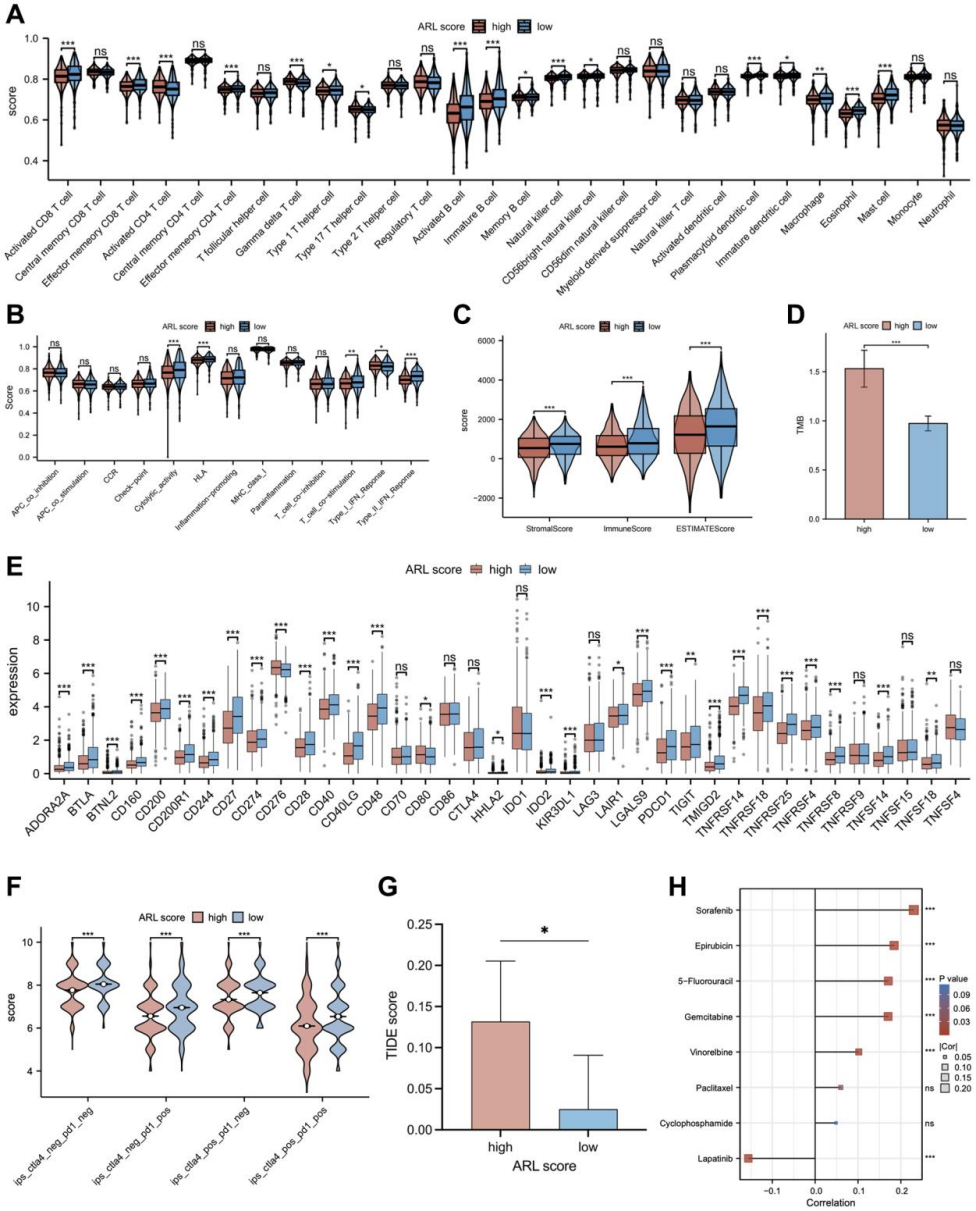
**The relationship between the ARL score and immune cell infiltration, immunotherapy response, and chemotherapy sensitivity.** (**A**) Comparison of immune cell infiltration in low versus high ARL score groups. (**B**) Comparison of enrichment of immune-related pathways between low versus high ARL score groups. (**C**) Comparison of immune and stromal scores between low versus high ARL groups. (**D**) Comparison of tumor mutation burden between low versus high ARL groups. (**E**) Comparison of expression levels of selected checkpoints between low versus high ARL score groups. (**F**) Comparison of immunotherapy response of PD-1 and CTLA4 between low versus high ARL score groups based on the results of immunogenomic analyses from The Cancer Immunome Atlas. (**G**) Comparison of immunotherapy response of PD-1 and CTLA4 between low versus high ARL score groups based on TIDE score. (**H**) Correlation between IC_50_ values of common chemotherapy drugs for breast cancer and ARL scores. Statistical significance symbols: ns, *p* ≥ 0.05; ^*^p < 0.05; ^**^p < 0.01; ^***^p < 0.001.

In light of mounting evidence that immune infiltration characteristics have a major impact on the progression and development of breast cancer, we accessed the relationship between immune infiltration characteristics and ARL scores. As shown in Figure 6A, the low ARL score group exhibited significantly higher infiltration fractions of immune cells (*P* < 0.05). Furthermore, Figure 6B demonstrates that immune-related pathways, including cytolytic activity, human leukocyte antigen (HLA), T cell co-stimulation, and type II IFN response, were highly enriched in the low ARL score group, whereas only the type I IFN response was highly enriched in the high ARL score group (*P* < 0.05). Additionally, the low ARL score group exhibited significantly higher immune, stromal, and estimated scores than the high ARL score group (*P* < 0.05), as depicted in Figure 6C.

To examine the relationship between ARL scores and the degree of immune cell infiltration in various breast cancer subtypes, we extended our analysis to include immune cell infiltration across five subtypes: basal-like/TNBC, HER2-enriched, luminal A, luminal B, and normal-like (Supplementary Figure 4). Despite the distinct immune cell infiltration characteristics among the four breast subtypes (basal-like/TNBC, HER2-enriched, luminal A, and normal-like), our data demonstrated that patients with low ARL scores invariably exhibited higher levels of immune cell infiltration (*P* < 0.05). Interestingly, patients with luminal B breast cancer deviated from this trend. An increase in the infiltration levels of certain immune cells, including central memory CD8 T cells, gamma delta T cells, type 17 T helper cells, CD56dim natural killer cells, and neutrophils, was significantly correlated with high ARL scores (*P* < 0.05). These findings underscore the fact that ARL scores can differentiate immune cell infiltration profiles among different breast cancer subtypes, which may aid the development of targeted treatments in the future.

### Immunotherapy response and chemotherapy sensitivity

Recently, immunotherapy and targeted therapy have played important roles in the clinical treatment of breast cancer and have become popular research topics. Therefore, a comparison was made between individuals with IBC who had low versus high ARL scores to determine the differences in responses to immune-targeted therapy. As shown in Figure 6E, most of the immune checkpoint candidate genes, such as *PD-L1*, were expressed at higher levels in the low ARL score group (*P* < 0.05). In addition, the results of immunogenomic analyses from The Cancer Immunome Atlas (TCIA) demonstrated that patients in the low ARL score group exhibited better responses to immunotherapy than patients in the high ARL score group, particularly when using CTLA4 targeted therapy alone, PD1 targeted therapy alone, or combined PD1 and CTLA4 targeted therapy, as well as targeted therapy excluding PD1 and CTLA4 (Figure 6F) (*P* < 0.05). Moreover, Tumor Immune Dysfunction and Exclusion (TIDE) analysis showed that patients in the low ARL score group had lower TIDE scores than patients in the high ARL score group, implying that those with low ARL scores may had better immunotherapy responses at PD-L1/CTLA4 immune checkpoint inhibitors (Figure 6G) (*P* < 0.05). Moreover, individuals with low ARL scores had lower tumor mutation burdens (TMBs) than patients with high ARL scores (Figure 6D) (*P* < 0.05). As chemotherapy plays an important role in the pre- and post-surgical treatment of patients with breast cancer, the role of the ARL score in chemotherapy for patients with IBC was explored in this study. Figure 6H illustrates the correlation between ARL scores and the half-maximal inhibitory concentration (IC_50_) of specific breast cancer chemotherapeutics. The IC_50_ values of chemotherapeutic drugs, including sorafenib, epirubicin, 5-fluorouracil, gemcitabine, and vinorelbine, were positively correlated with the ARL score (*P* < 0.05), whereas that of lapatinib was negatively correlated with the ARL score (*P* < 0.05). These findings suggest that individuals with different ARL scores may benefit from appropriate chemotherapeutic drugs tailored to their ARL, thereby potentially improving the prognosis of patients with IBC.

### Validation of the expression levels of the 7 prognostic LncRNAs

To analyze the expression profiles in clinical samples, we investigated the expression levels of the seven core prognostic lncRNAs in both TCGA cohort and clinical samples. As depicted in Supplementary Figure 5A, 5B, *C60rf99, LINC01614, AC004585, MAPT-IT1*, and *AC004585.1* exhibited significantly higher expression levels in breast tumor tissues than in paired or unpaired normal breast tissues (*P* < 0.05). In contrast, *LINC02613* and *AL133467.1* displayed significantly lower expression levels in breast tumor tissues than in paired or unpaired normal breast tissues (*P* < 0.05). Additionally, quantitative reverse transcription-polymerase chain reaction (qRT-PCR) analysis revealed that the expression levels of *C60rf99, LINC02613, AL133467.1, MAPT-IT1*, and *AC004585.1* were downregulated in breast tumor tissues relative to normal breast tissues (*P* < 0.05) (Supplementary Figure 5C). However, no significant difference was observed in the expression levels of *LINC01614* and *AC055854.1* between tumor and normal clinical samples (*P* > 0.05) (Supplementary Figure 5C).

### Effect of *AL133467.1* expression in pan-cancer

The expression levels of *AL133467.1* were investigated across a range of cancers by contrasting tumorous and corresponding normal tissues. Notably, *AL133467.1* demonstrated elevated expression in lung adenocarcinoma (LUAD) and lung squamous cell carcinoma (LUSC) (*P* < 0.05). In stark contrast, *AL133467.1* displayed significantly reduced expression in colon adenocarcinoma (COAD), colon adenocarcinoma/rectum adenocarcinoma esophageal carcinoma (COADREAD), invasive breast carcinoma (BRCA), kidney renal papillary cell carcinoma (KIRP), liver hepatocellular carcinoma (LIHC), thyroid carcinoma (THCA), rectum adenocarcinoma (READ), bladder urothelial carcinoma (BLCA), kidney chromophobe (KICH), and cholangiocarcinoma (CHOL) (*P* < 0.05) as depicted in Supplementary Figure 6A). The expression of *AL133467.1* did not differ significantly between normal and tumorous tissues in the other 14 cancer types.

Survival analyses were conducted for each cancer type to assess the role of *AL133467.1* in the prognosis of patients with different cancer types. A univariate Cox analysis revealed a significant association between *AL133467.1* levels and overall survival (OS) outcomes in pan-kidney (KIPAN; *P* = 3.8e-4), glioblastoma multiforme (GBM; *P* = 0.05), kidney renal clear cell carcinoma (KIRC; *P* = 0.03), COADREAD (*P* = 0.03), KICH (*P* = 6.8e-5), BRCA (*P* = 0.01), head and neck squamous cell carcinoma (HNSC; *P* = 0.02), skin cutaneous melanoma (SKCM; *P* = 0.02), SKCM-M (*P* = 0.02), and ovarian serous cystadenocarcinoma (OV; *P* = 2.3e-3), as shown in Supplementary Figure 6B. These findings suggest that *AL133467.1* could be considered to be a high-risk gene in KIPAN, GBM, KIRC, COADREAD, and KICH but a low-risk gene in BRCA, HNSC, SKCM-M, SKCM, and OV.

We employed an additional correlation analysis to elucidate the relationship between *AL133467.1* expression levels and immune-related markers across diverse types of cancer. As depicted in Supplementary Figure 6C, *AL133467.1* manifested positive correlation with both immune inhibitory and stimulatory markers across most cancer types. The correlation between *AL133467.1* expression and various cell infiltration levels within the tumor microenvironment was subsequently investigated. Our findings demonstrate that an increase in *AL133467.1* expression considerably amplified the infiltration levels of numerous cells within the microenvironment across most tumor types. As indicated in Supplementary Figure 6D, cells such as aDC, B cells, CD4+ memory T cells, CD4+ naive T cells, CD8+ T cells, CD8+ Tcm, CD8+ Tem, cDC, chondrocytes, class-switched memory B cells, DC, hematopoietic stem cells (HSCs), iDC, M2 macrophages, M1 macrophages, mast cells, megakaryocytes, melanocytes, memory B cells, monocytes, multipotent progenitor (MPP) cells, endothelial cells, naive B cells, pDCs, plasma cells, and Tgd cells demonstrated a positive correlation with heightened *AL133467.1* expression in most cancer types (*P* < 0.05). In contrast, common lymphoid progenitor (CLP) cells, osteoblasts, epithelial cells, and smooth muscle cells were negatively associated with *AL133467.1* expression in most cancer types (*P* < 0.05). In addition, scores pertaining to the immune response, stromal components, and overall tumor microenvironment were positively correlated with *AL133467.1* expression. These results underscore the strong association between *AL133467.1* and the tumor microenvironment across an array of cancer types.

### Elevated expression of *AL133467.1* notably reduced the proliferation and migration of breast cancer cells

**Figure 7:**
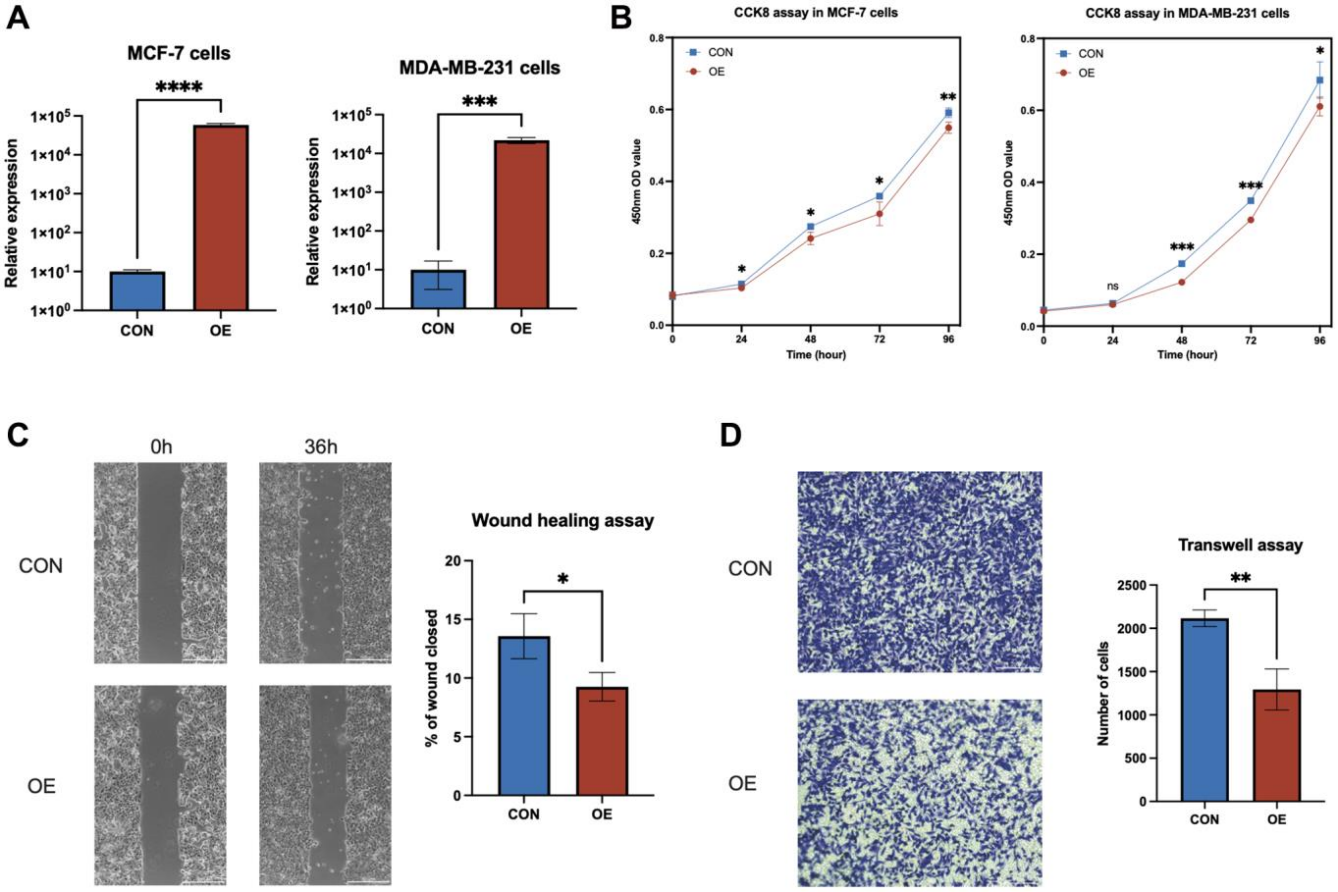
***In vitro* experiments to investigate the role of *AL133467.1* in breast cancer cells.** (**A**) qRT-PCR results showing the overexpression of *AL133467.1* in MCF-7 and MDA-MB-231 cells. (**B**) CCK-8 assay results of MCF-7 and MDA-MB-231 cell proliferation. (**C**) Wound-healing assay results evaluating MCF-7 cell migration. (**D**) Transwell assay results of MDA-MB-231 cell migration. Statistical significance symbols: ns, *p* ≥ 0.05; ^*^p < 0.05; ^**^p < 0.01; ^***^p < 0.001.

To decipher the molecular roles of anoikis-related genes (ARGs) in breast cancer, a comprehensive study on *AL133467.1*, based on findings from qRT-PCR and survival analyses, aimed to clarify its distinct function in IBC. Transient transfection was carried out for *AL133467.1*, to attain its overexpression in both MCF-7 and MDA-MB-231 cell lines (Figure 7A). CCK-8 assay confirmed that enhanced expression of *AL133467.1* greatly reduced the proliferation of both MCF-7 and MDA-MB-231 cells (Figure 7B). Moreover, a wound-healing assay showed that enhanced expression of AL133467.1 significantly reduced the migration potential of MCF-7 cells (Figure 7C). Lastly, Transwell assay demonstrated that enhanced expression of *AL133467.1* significantly reduced the migration of MDA-MB-231 cells (Figure 7D).

### Over-expression of *AL133467.1* impaired anoikis in breast cancer cells

**Figure 8:**
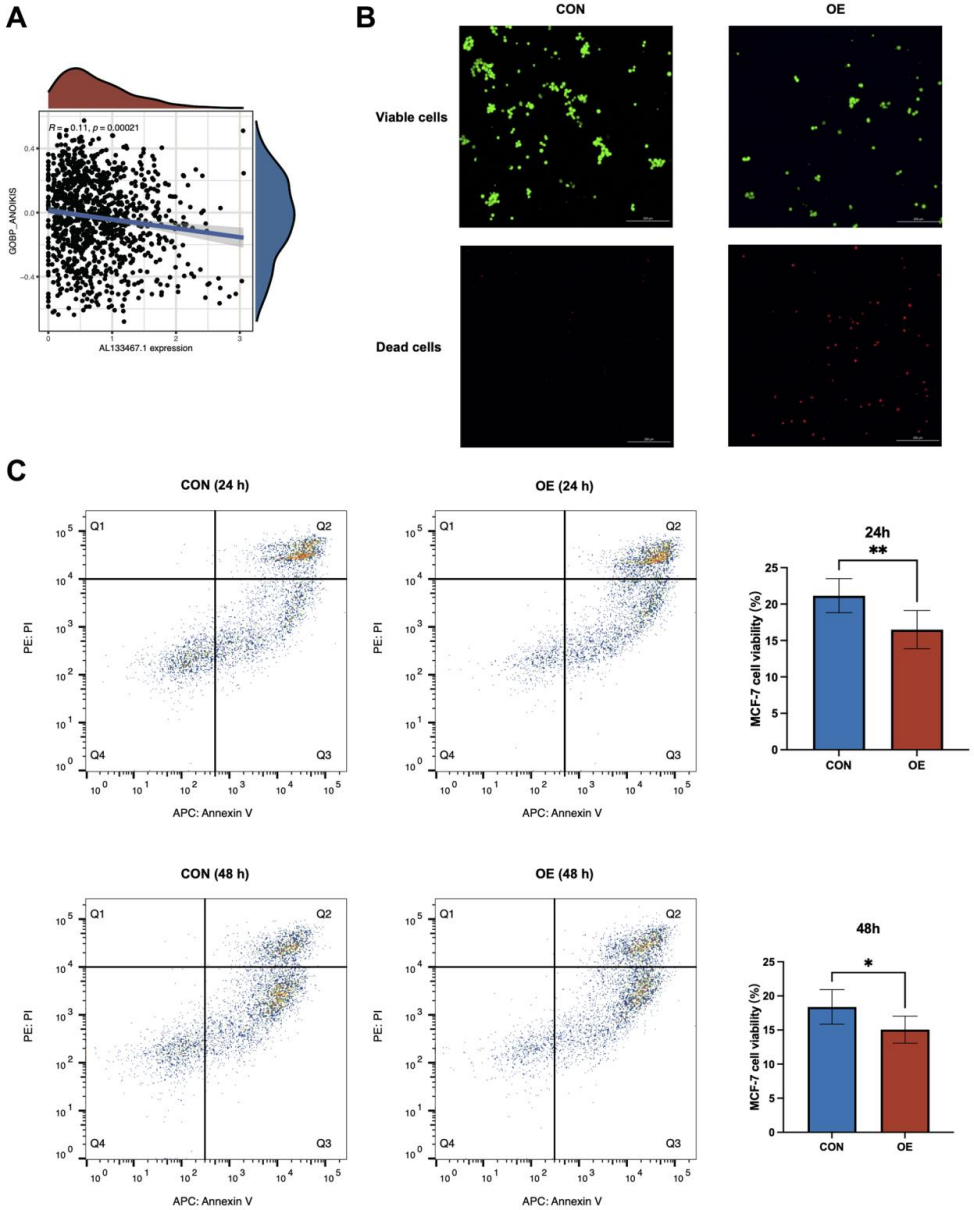
***In vitro* experiments to investigate the relationship between *AL133467.1* and anoikis in breast cancer cells.** (**A**) Correlation analysis between *AL133467.1* expression and anoikis. (**B**) Fluorescence detection to explore the role of *AL133467.1* in anoikis regulation in MCF-7 cells; Green: viable cells; Red: dead cells. (**C**) Quantitative analysis of the effect of *AL133467.1* on anoikis using flow cytometry. Statistical significance symbols: ns, *p* ≥ 0.05; ^*^p < 0.05; ^**^p < 0.01; ^***^p < 0.001.

To elucidate the effect of *AL133467.1* expression on anoikis in breast cancer, we studied the association between *AL133467.1* expression and anoikis. As depicted in Figure 8A, *AL133467.1* expression was negatively correlated with anoikis, suggesting that *AL133467.1* can potentially function as a negative regulator of anoikis. To confirm these findings, we induced anoikis in MCF-7 cells by preventing cell adhesion to the culture dish. As depicted in Figure 8B, following a 24-hour incubation, the cells overexpressing *AL133467.1* exhibited lower viability than the control cells. To quantitatively assess differences between the two groups, flow cytometry was used to measure cell viability. After a 24-hour or 48-hour incubation in an anti-adhesion environment, the overexpression group displayed significantly reduced cell viability relative to the control group (Figure 8C). These findings suggest that overexpression of *AL133467.1* may enhance anoikis in MCF-7 cells.

## DISCUSSION

Anoikis is a unique variant of apoptosis that has significant physiological relevance in organisms as it circumvents unwarranted cell adherence and subsequent proliferation [14]. Recent research has highlighted the pivotal role of anoikis in breast cancer ontogeny and metastasis. Many studies have emphasized that the counter-anoikis signal is a critical phase for breast cancer cells to obtain the capability of invasion and metastasis [15]. Breast cancer cells survive within the circulatory system by impeding anoikis, thereby increasing the likelihood of establishing colonies in distal tissues. Consequently, exploring the mechanisms inhibiting anoikis and methods to obstruct these processes has become a key area for future breast cancer research [4]. Moreover, anoikis could potentially serve as a novel target for drug discovery. For instance, medications designed to counteract anoikis resistance mechanisms may bolster treatment efficacies in patients with breast cancer [16]. To the best of our knowledge, previous studies have not systematically evaluated the prognostic value of anoikis-associated lncRNAs in breast cancer. Thus, this study can be used as the main reference for subsequent research.

LncRNAs are important for the progression of breast cancer. Specific lncRNAs have been reported to either promote or hinder breast cancer progression through key processes that influence tumor proliferation, invasion, and metastasis [17]. For instance, the lncRNA *HOTAIR*, which is overexpressed in breast cancer, can change gene expression through alterations in chromosome structure [18]. In contrast, expression of the lncRNA *GAS5* is diminished in many forms of breast cancer, and its loss is associated with the incidence and progression of breast cancer [19]. These findings indicate that lncRNAs may exert a significant effect on the pathogenesis of breast cancer, thus providing a potential novel approach for breast cancer treatment.

We established and validated a signature of seven ARLs to predict the prognosis of breast cancer patients. The OS of patients in the low and high ARL score groups was distinctly stratified using ARL signatures. Five ARLs (*MAPT.IT1, AL133467.1, AC004585.1, AC055854.1*, and *LINC02613*) were identified as protective factors, whereas two (*LINC01614* and *C6orf99*) were identified as risk factors for breast cancer. *In vitro* functional experiments indicated a significant role of *AL133467.1* in the proliferation and migration of breast cancer cells, as well as a certain degree of impact on the resistance of breast cancer cells to anoikis. Previous studies have not reported the role of *AL133467.1* in breast cancer; thus, exploring the relationship between *AL133467.1* and anoikis could potentially aid in understanding *AL133467.1*’s role in breast cancer.

In addition, we investigated the correlation between ARL scores and clinicopathological features. Among the parameters of age, tumor stage (T), node stage (N), metastatic stage (M), pathological stage, and ARL score, only age and the ARL score were identified as independent prognostic factors for IBC in the complete training and test sets. This underscores the ability of the ARL score to independently predict the prognosis of IBC. To enhance its clinical applicability, we integrated the ARL score with other clinical prognostic factors to construct a prognostic nomogram. The ROC curve, calibration curve, and DCA confirmed that the nomogram exhibited considerable advantages in predicting survival and guiding clinical decision-making compared with traditional clinicopathological features.

In this study, the results of GSVA enrichment analysis revealed that patients with elevated ARL scores demonstrated significant activation of cell cycle, energy metabolism, and biosynthesis-related pathways, including the proteasome, cell cycle, DNA replication, homologous recombination, and mismatch repair. Increased activation of these pathways may facilitate uncontrolled cellular proliferation and tumorigenesis [20–23]. Concurrently, the amplified expression of energy metabolism pathways such as glycolysis, the citrate cycle (TCA cycle), oxidative phosphorylation, and the pentose phosphate pathway may satisfy the energy requirements of tumor cells [24, 25]. Furthermore, an increase in the expression of amino sugars and nucleotide sugar metabolism, folate biosynthesis, terpenoid backbone biosynthesis, and steroid biosynthesis can promote the growth of tumor cells [26–28]. In contrast, patients with lower ARL scores exhibit increased expression of immune response-related pathways such as primary immunity, hematopoietic cell lineage, and an intentional immune network for IgA production, reflecting the potential protective role of a robust immune system in averting tumorigenesis [29, 30]. In addition, upregulation of circadian rhythms in mammals may underscore the tumor-suppressive role of a well-functioning biological clock [31]. Although these preliminary analyses contributed to a comprehensive understanding of the prognostic implications of ARL scores in patients with breast cancer, there is a compelling need for further research to corroborate and expand these observations.

Gene mutations, particularly in pivotal tumor suppressor genes and oncogenes, can induce unregulated cell growth and division, thereby promoting tumor formation. These alterations can modulate sensitivity to specific treatments and provide critical information for clinical decision-making and prognostic prediction [32]. In our analysis, several key genes, such as *TP53* and *PIK3CA*, exhibited diverse mutation frequencies in breast cancer patients with high and low ARL scores. Intriguingly, we discovered a significantly higher mutation frequency in *TP53* among patients with poor prognoses (47%) than among those with good prognoses (20%). Given the crucial role of TP53 as a tumor suppressor, its mutation may precipitate unregulated cell growth and division, thereby increasing tumor risk [33]. Hence, the elevated TP53 mutation rate may suggest enhanced tumorigenic potential in breast cancer patients with a high ARL score. Concurrently, we observed a higher mutation frequency in PIK3CA in patients with a low ARL score (44%) than in those with a high ARL score (26%). The protein encoded by *PIK3CA* plays a pivotal role in various biological processes including cell growth, survival, and metabolism [34]. While the reason for the higher PIK3CA mutation frequency in patients with a better prognosis remains elusive, it may suggest novel therapeutic avenues, such as PIK3CA-specific mutation inhibitors. Additionally, we detected high mutation frequencies in TTN and GATA3 across both the high and low ARL score groups, potentially reflecting common biological characteristics of breast cancer and underscoring their importance in breast cancer pathogenesis [35, 36]. Although our research has provided interesting insights, various factors warrant further exploration, including the type and location of mutations and the impact of other genetic and environmental factors on breast cancer prognosis.

To delve deeper into the function of the prognostic model based on the ARL score in IBC, we explored the correlation between chemotherapy and ARL scores. Lapatinib, vinorelbine, gemcitabine, 5-fluorouracil, epirubicin, and sorafenib are used for breast cancer chemotherapy. Our results demonstrated a positive association between the IC_50_ values of vinorelbine, gemcitabine, 5-fluorouracil, epirubicin, and sorafenib and the ARL score, whereas a negative relationship was found between the IC_50_ value of lapatinib, indicating a strong link between the ARL score and chemotherapy. This discovery further highlights the potential of the prognostic model and may offer clinical guidance for the selection of chemotherapeutic drugs for breast cancer patients with different ARL scores.

Immune cell dynamics within the tumor microenvironment play a pivotal role in determining breast cancer prognoses [37]. Our results revealed that patients with low ARL scores exhibited significantly increased infiltration of activated CD8 T cells, effector memory CD8 T cells, effector memory CD4 T cells, and type 1 T helper cells. These cells are integral to tumor inhibition, especially in facilitating cytotoxic reactions to eliminate tumor cells [38]. Additionally, we observed substantial infiltration of natural killer cells into the tumor microenvironment of patients with low ARL scores. As natural antitumor immune cells, natural killer cells play a critical role in directly eliminating tumor cells and orchestrating adaptive immune responses [39]. In contrast, patients with high ARL scores had elevated levels of activated CD4 T cells, gamma-delta T cells, and type 17 T helper cells. This may reflect an immunosuppressive phenomenon within the tumor microenvironment, and these cells can contribute to the regulation of inflammation and the survival of tumor cells during tumorigenesis [40–42]. Our findings underscore the importance of in-depth examination of the roles of these immune cell subsets in breast cancer prognosis, which has profound implications for devising more effective antitumor immunotherapies.

Despite these insightful findings, this study has several limitations. First, prospective multicenter studies with extensive BRCA cohorts are warranted to corroborate the reliability of the ARL signature and the accompanying results. Secondly, although we explored the functionality of one ARL *in vitro*, additional experimental investigations are necessary to elucidate its regulatory mechanisms and functions.

## MATERIALS AND METHODS

### Data collection

**Figure 9:**
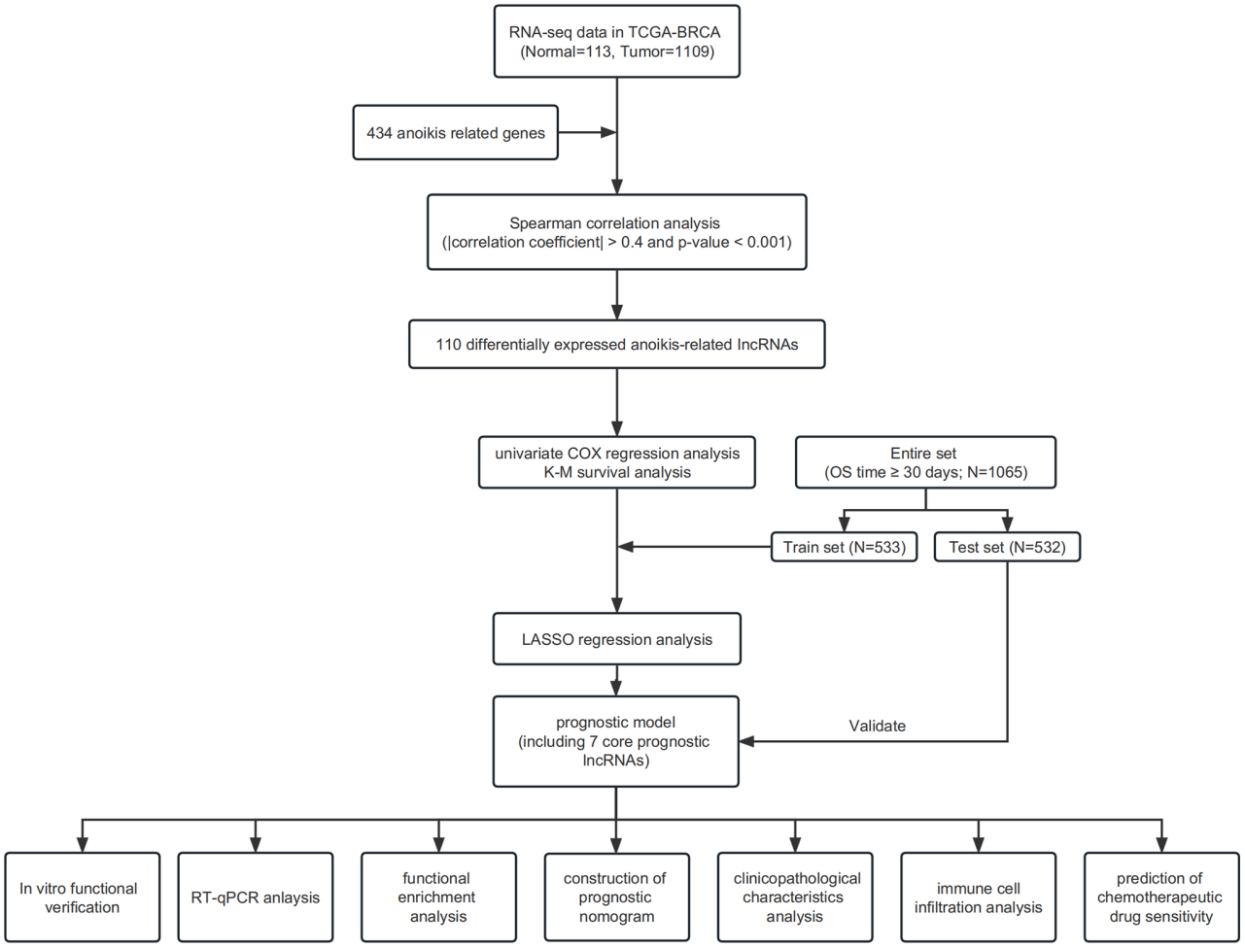
Flowchart.

The main information utilized for this research was obtained from TCGA repository, comprising 1,109 IBC samples and 113 healthy breast tissue samples. Patients with unknown clinical information and an overall survival time of less than 30 days were excluded from the study. Gene sets associated with anoikis were obtained from a prior investigation [43]. Figure 9 shows the flowchart of our study.

### Identification of anoikis-related differentially expressed lncRNAs

The package “DESeq2” was utilized to screen DELs between IBC versus normal breast tissues in TCGA dataset [44]. DELs were identified based on a log2 (fold change) >1 and an adjusted *p*-value < 0.05. Spearman’s analysis was used to investigate the relationship between lncRNAs and ARGs. To identify anoikis-related lncRNAs, we used a previously described co-expression gene identification method [45, 46]. This method involves the selection of lncRNAs based on two criteria: a Spearman correlation coefficient greater than 0.4 and a *p*-value below 0.001. Anoikis-related DELs were obtained by intersecting DELs with anoikis-related lncRNAs.

### Prognostic model construction and verification

The RNA data of anoikis-related DELs and clinical information on IBC from TCGA were combined based on the sample ID. Only patients with an OS time of 30 days or more were included in further survival analyses. Univariate Cox regression analysis was performed using the Kaplan–Meier “survival” package [47]. Subsequently, for further identification of the core genes related to anoikis and the construction of an ARL score-based prognostic model, we employed the “glmnet” and “survival” packages to conduct LASSO logistic regression analysis [48]. The following formula was used to calculate the ARL score of IBC patients: *C6orf99* expression value × corresponding coef + *LINC01614* expression value × corresponding coef + *LINC02613* expression value × corresponding coef + *AC055854.1* expression value × corresponding coef + *AL133467.1* expression value × corresponding coef + *AC004585.1* expression value × corresponding coef + *MAPT.IT1* expression value × corresponding coef. The Shapiro–Wilk normality test and Wilcoxon test were used to perform a difference analysis between the two groups. The Spearman method was used to perform correlation analysis. Kaplan–Meier survival analysis was performed using the “survminer” and “survival” packages [47]. PCA was conducted using the “factoextra” and “FactoMineR” packages [49]. ROC analysis was performed using the “timeROC” package [47].

### Correlation between clinical characteristics and the prognostic model

We used the Wilcoxon test to evaluate the differences in ARL scores among patients with different clinicopathological features. Correlation analysis was performed using the Spearman method. The “maftools” package was utilized to explore the somatic variants between individuals with low and high ARL scores [50].

### Construction of the nomogram for predicting the prognosis of IBC

Univariate Cox analysis was carried out using the “survival” package [47]. The calibration analysis and nomogram construction were carried out using the “rms” and “survival” packages [47]. The “timeROC” package was employed for ROC analysis [47]. The decision curve analysis (DCA) was carried out using the “survival” and “survminer” packages [51].

### Function enrichment analysis

We conducted Gene Ontology (GO) and Kyoto Encyclopedia of Genes and Genomes (KEGG) enrichment analyses using the “clusterProfiler” package [52]. Gene set enrichment analysis (GSEA) was performed using the GSEA software available on the GSEA website (http://software.broadinstitute.org/gsea/index.jsp) and the “h.all.v2022.1. Hs.symbols.gmt” gene sets were sourced from the Molecular Signatures Database. Furthermore, Gene Set Variation Analysis (GSVA) was carried out using the “GSEABase” and “GSVA” packages, along with the “c2.cp.kegg.v2022.1. Hs.symbols.gmt” gene set [53, 54]. A false discovery rate (FDR) of less than 0.25 and a *P*-value cutoff of less than 0.05 were used to evaluate statistical significance.

### Immune infiltration characteristics of the TME

The analysis of immune cell infiltration and immune-related pathway enrichment was conducted using the “GSVA” and “GSEABase” packages [53, 54]. Marker genes of immune cells identified by Bindea et al. [54] were used in this analysis. Moreover, we employed the “estimate” package to calculate immune and stromal scores in both the low ARL score group and the high ARL score group. [55]. The Shapiro–Wilk normality test and Wilcoxon test were used to examine the differences between groups.

### Immunotherapy response and chemotherapy sensitivity

We retrieved immunogenomic examination data from TCIA website (https://tcia.at/home) [56]. We used TIDE (http://tide.dfci.harvard.edu/) to evaluate the predictive effect of anoikis-related lncRNAs signatures on immunotherapy response. We used the Shapiro–Wilk normality test and Wilcoxon test to assess the disparity between the two clusters. The evaluation of drug susceptibility was executed by applying the “oncopredict” package [57]. We employed the Spearman approach for correlation analysis.

### qRT-PCR

The study utilized tissue samples from the biobank of Shanghai First Maternity and Infant Hospital and was conducted with the exemption of informed consent by the Ethics Committee of the same hospital. Total RNA was obtained from 15 breast cancer samples and 12 nearby normal breast tissues using Total RNA Extraction Reagent (TRIzol^®^) (RK30129, ABclonal, USA), according to the guidelines provided by the manufacturer. With the assistance of ABScript III RT Master Mix (RK20428, ABclonal), we performed the reverse transcription using 1 μg of total RNA. qRT-PCR analysis was conducted utilizing Genlous 2× SYBR™ Green PCR Fast qPCR Mix (Low ROX Premixed) (RK21206, ABclonal). The evaluation of the comparative expression of the genes of concern was carried out utilizing the 2^−ΔΔCT^ methodology, with beta-actin serving as the reference gene. The primer sequences used are listed in Supplementary Table 1.

### Pan-cancer analysis

To conduct a pan-cancer differential analysis of *AL133467.1*, we obtained a standardized and consolidated dataset called TCGA Pan-Cancer (Pancan, *N* = 10535, G = 60499) from the UCSC database (https://xenabrowser.net/). From this dataset, we specifically extracted the expression data of *AL133467.1* for every individual sample. Samples were selected specifically from healthy solid tissues and cancerous peripheral blood derived from primary tumors. Samples with no expression levels were excluded, and all expression values were converted using the log2(x+1) transformation. The expression data for 26 cancer types were obtained after excluding those with fewer than three samples. The Wilcoxon test was used to determine the differences between two groups.

For the survival analysis, in addition to obtaining survival data from the UCSC, we procured a high-quality prognostic dataset from TCGA using an integrated TCGA pan-cancer clinical data resource [47]. Samples with zero expression levels were excluded, as were samples with a follow-up time of less than 30 days. All expression values were transformed by log2(x+1). Cancers with fewer than 10 samples were excluded, and 39 cancer types were included (Supplementary Figure 6B). We employed the coxph function of the R software survival package to perform the Cox regression analysis [58]. The significance of the prognosis was determined through a statistical test with the Logrank test.

To analyze immune checkpoint markers, we obtained an identical comprehensive pan-cancer dataset from the UCSC database. From each sample, we extracted the expression of 60 genes related to immune checkpoint pathways, consisting of 24 inhibitory and 36 stimulatory genes [59]. The association between *AL133467.1* and immune checkpoint marker genes was investigated by Pearson correlation analysis.

For immune infiltration analysis, we extracted the gene expression profiles of each cancer type from the UCSC pan-cancer dataset, mapped the expression profiles to the gene symbol, and employed the R software package “IOBR” [60] and deconvo_xCell method [61] to estimate the infiltration scores of 67 tumor microenvironment cell types in 39 cancer types, based on gene expression data. The “psych” package was used to examine the relationship between *AL133467.1* and levels of immune cell infiltration in various tumor types.

### Cell culture and plasmid transfection

The human breast cancer cell lines MCF-7 and MDA-MB-231 were sourced from Wuhan Procell Life Science and Technology Co., Ltd., (Wuhan, China). We propagated MCF-7 cells in minimum Eagle’s medium (MEM), supplemented with 10% fetal bovine serum (Gibco, USA), 0.01 mg/mL insulin, 100 U/mL penicillin G, and 100 μg/mL streptomycin. MDA-MB-231 cells were grown in Dulbecco’s modified Eagle’s medium (DMEM), supplemented with 10% fetal bovine serum (Gibco, USA), 100 U/mL penicillin G, and 100 μg/mL streptomycin. Both cell lines were incubated in a 5% CO_2_ atmosphere at 37°C. To assess the effect of *AL133467.1*, Lipofectamine™ 3000 (Invitrogen, USA) was used to transfect pcDNA-*AL133467.1*/pcDNA (negative control) into MCF-7 and MDA-MB-231 cells according to the Lipofectamine™ 3000 reagent protocol. Functional assays were performed, and RNA was collected 48 h after transfection.

### Cell phenotype assays

The proliferative behavior of breast cancer cells was investigated by CCK-8 assay. Following digestion, cells from different treatment sets were seeded into 96-well plates (Corning, USA) at a cell density of 2,000 cells/well. The CCK-8 assay was performed as per the manufacturer’s guidelines, and the absorbance was measured at 450 nm using a microplate reader.

To examine the migratory capacity of breast cancer cells, wound-healing and Transwell assays were performed. For the wound-healing assay, digested cells from various treatment groups were placed in a Culture-Insert 2 Well (Ibidi, Germany) in 24-well plates (Corning, USA). Following a 24-hour incubation period, the Culture-Insert 2 Well was meticulously removed, generating a cell-free zone of roughly 500 μm. An inverted microscope (Nikon Ts2R, Japan) at 40× magnification was used to capture images of the gap area at 0 and 36 h.

For the Transwell assay, 800 μL of medium supplemented with 10% fetal bovine serum (Gibco) was deposited in the lower chamber of a Transwell™ insert, and the upper chamber was filled with 200 μL of serum-free medium containing 50,000 cells. After a 48-hour incubation phase, cells that had crossed the membrane were fixed using 4% paraformaldehyde, rinsed with phosphate-buffered saline, stained with 0.3% crystal violet, and imaged with an inverted microscope at 100× magnification.

### Anoikis assay

Cells from different treatment groups were digested and seeded in anchorage-resistant 96-well plates of an Anoikis Assay Kit (Abcam, UK) at a density of 2,000 cells per well to simulate the state of anoikis. After 24 h of incubation, the culture medium was supplemented with calcein AM and ethidium homodimer from the Anoikis Assay Kit (Abcam, UK), followed by an additional incubation period of 30–60 min. After observing the cells under a fluorescence microscope, fluorometric signals were detected. For flow cytometric analysis, cells from the various treatment groups were digested and seeded in ultra-low-attachment 6-well plates (Corning, USA) at a density of 100,000 cells/well. Following incubation for 24 or 48 h, the cells from the respective treatment groups were collected and subjected to flow cytometry to detect apoptosis. This analysis was performed using an Annexin V-Alexa Fluor 647/PI Apoptosis Detection Kit (Yeasen, China) following the manufacturer’s instructions.

### Statistical analysis

The process of data analysis and visual representation was executed using R software (versions 3.6.3 and 4.2.1) and GraphPad Prism software (version 9.0). Unless otherwise indicated, the Wilcoxon test was utilized to discern differences between two groups, the Spearman test was employed to probe correlations between two groups, and Kaplan–Meier survival analysis was applied to evaluate survival differences between two sets. Statistical significance was set at *p* < 0.05.

## CONCLUSION

Our study constructed and validated a suite of lncRNA models based on ARL scoring, thereby offering a powerful prognostic tool for breast cancer. Furthermore, a comprehensive nomogram was created employing both clinicopathological parameters and the ARL score to predict overall survival in a clinical setting. We demonstrated a significant inverse relationship between TP53 and PIK3CA mutation frequencies relative to the ARL score. Intriguingly, pathways associated with the cell cycle, energy metabolism, and biosynthesis demonstrated increased activation in the high ARL score group. We found a robust correlation between the ARL score and the extent of immune cell infiltration, TMB, expression of immune checkpoints, and predictive IC_50_ values of chemotherapy agents. Moreover, lncRNA *AL133467.1* affected both breast cancer cell proliferation and migration, as well as possibly resistance to anoikis. In conclusion, the ARL signature, as a holistic prognostic tool, facilitates the assessment of risk stratification and informs the clinical decision-making processes for patients with breast cancer. Further investigation is required to elucidate the broader clinical implications of these findings.

## Supplementary Materials

Supplementary Figures

Supplementary Table 1
