# Supplementary material for: Deciphering the prognostic significance of anoikis-related lncRNAs in invasive breast cancer: from comprehensive bioinformatics analysis to functional experimental validation
Source: Aging (Albany NY). 2024 Jan 5;16(1):402–30. doi: 10.18632/aging.205376 (PMC10817393; doi:10.18632/aging.205376)
Supplement: Supplementary Figures [file aging-16-205376-s001.pdf]

SUPPLEMENTARY FIGURES

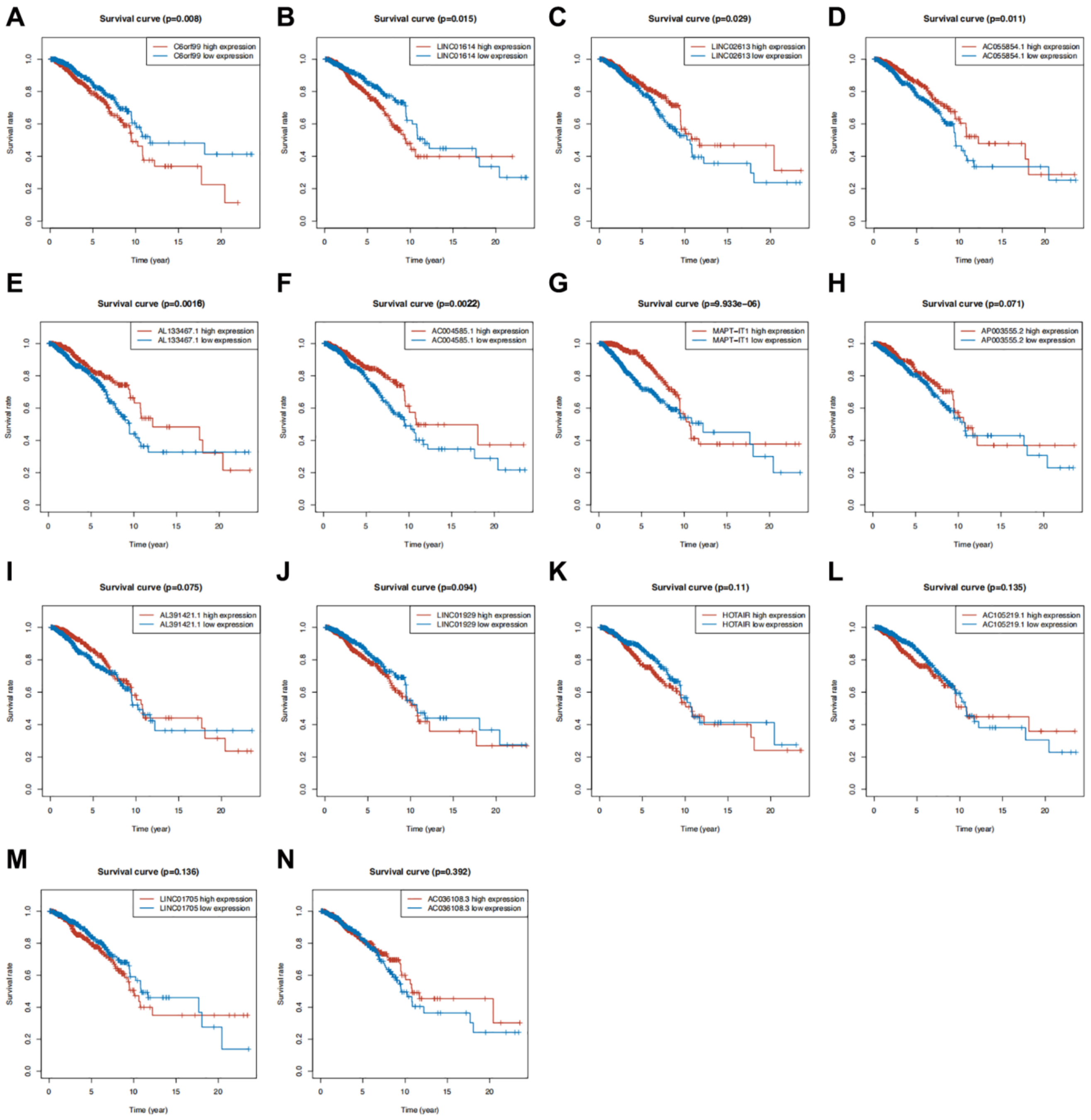

Supplementary Figure 1. Kaplan–Meier survival analysis results of 14 prognostic lncRNAs.

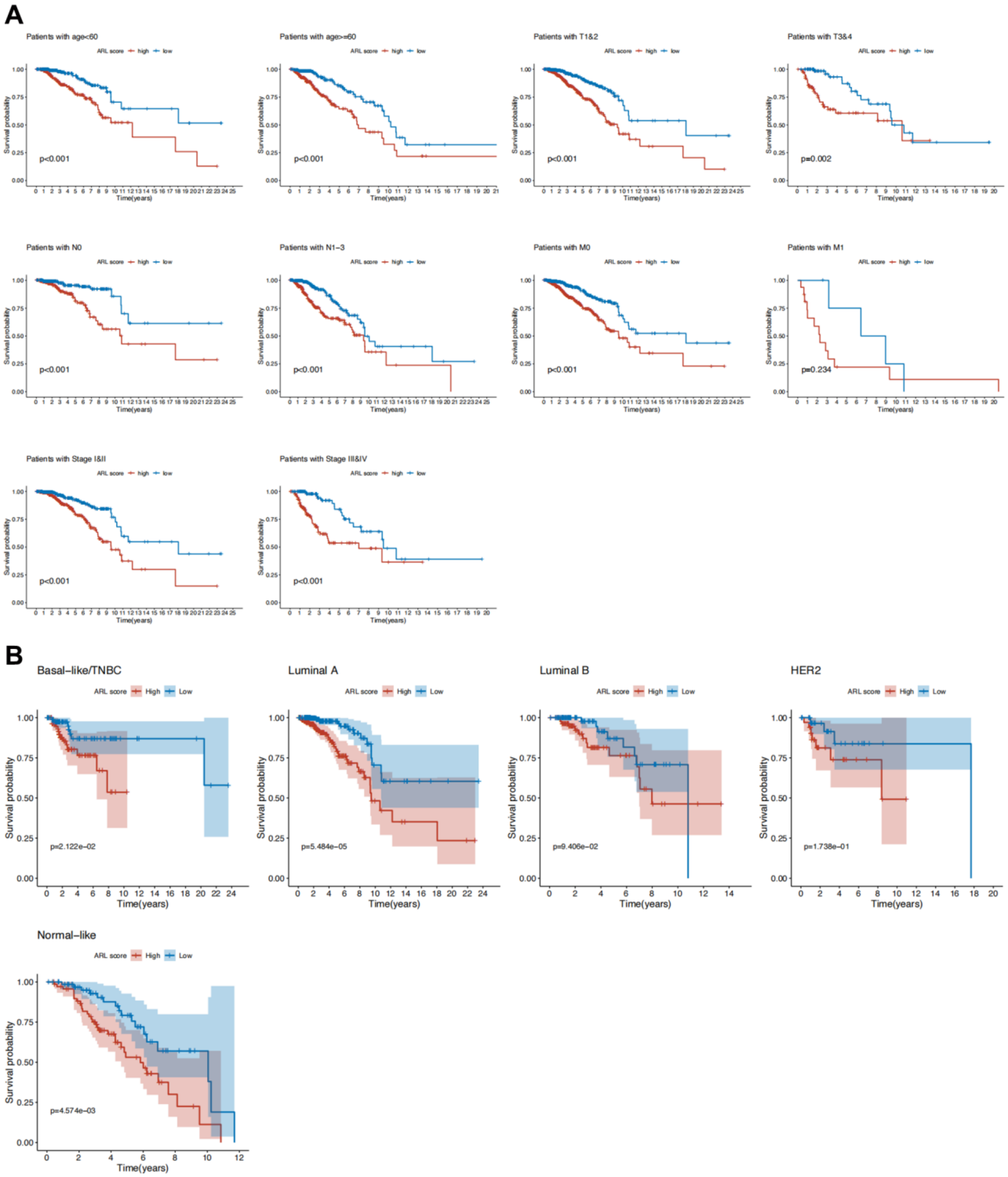

Supplementary Figure 2. Survival analysis of patients with high and low ARL scores across various clinicopathological characteristics in the entire set. (**A**) Kaplan–Meier survival analysis stratified by age, T stage, N stage, M stage, and pathological stage. (**B**) Survival analysis across PAM50 molecular subtypes of breast cancer.

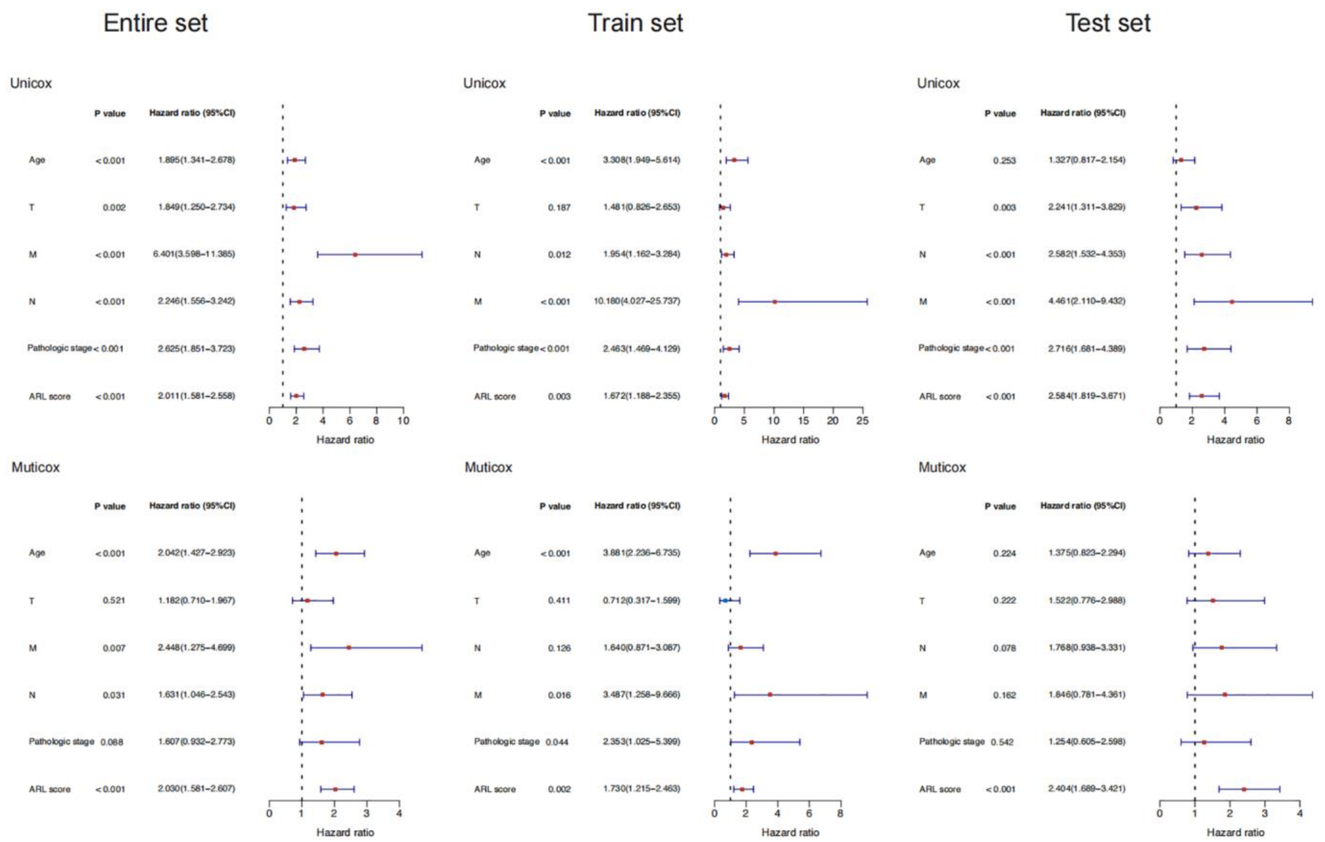

Supplementary Figure 3. Univariate and multivariate Cox regression analyses of clinical characteristics and ARL scores.

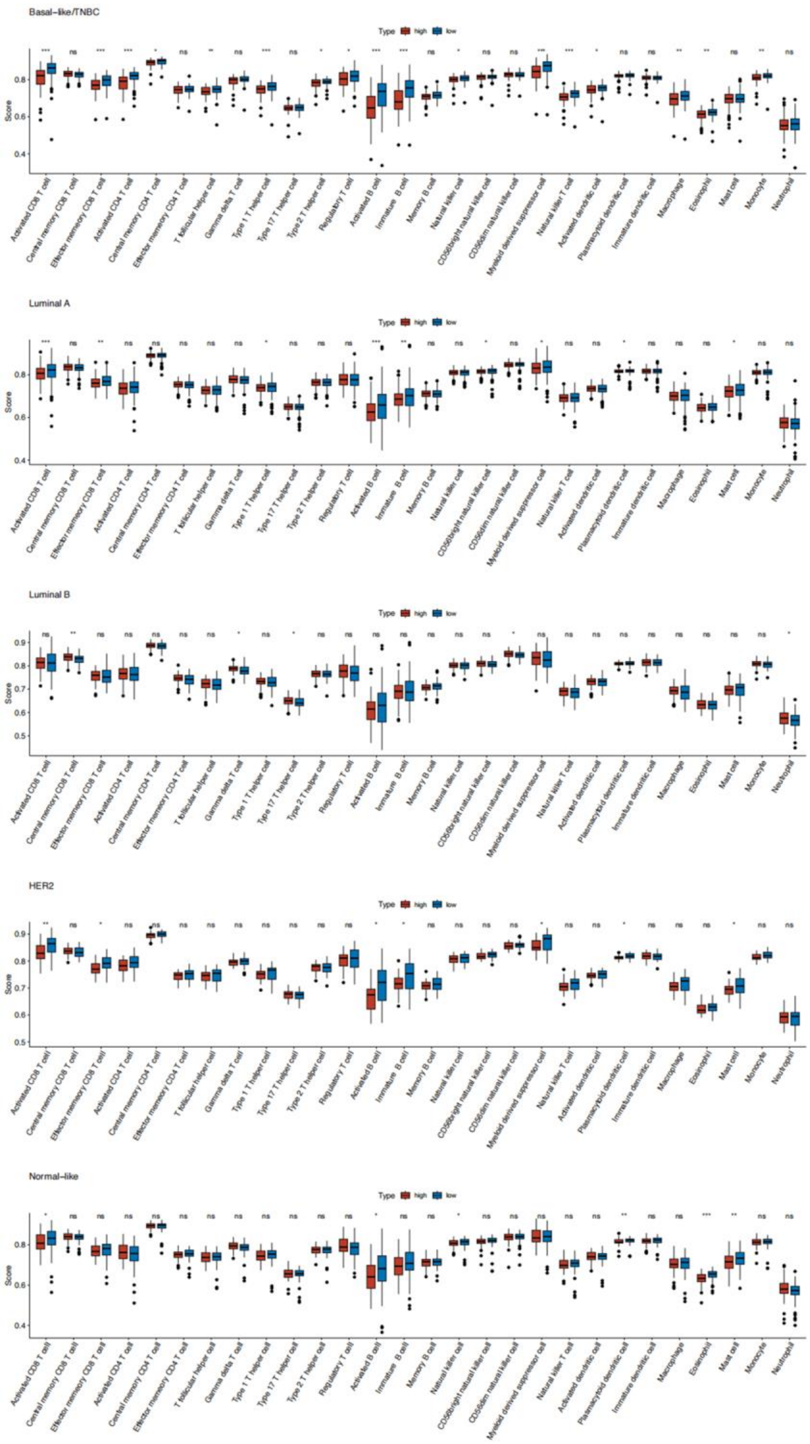

Supplementary Figure 4. The relationship between the ARL score and immune cell infiltration in breast cancer subtypes. Statistical significance symbols: ns, $p \geq 0.05$; $^{*}p < 0.05$; $^{**}p < 0.01$; $^{***}p < 0.001$.

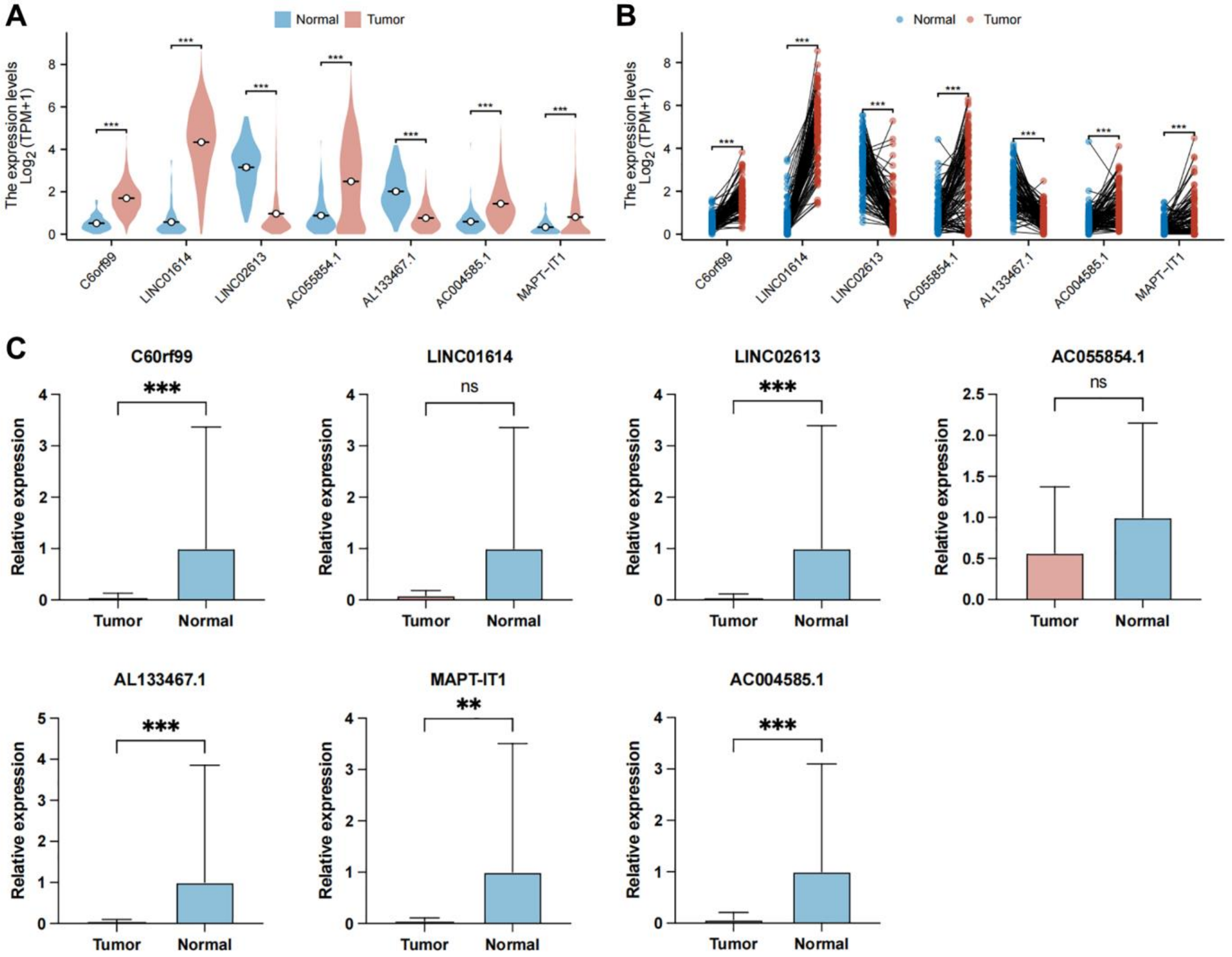

Supplementary Figure 5. Expression levels of the seven core prognostic lncRNAs in normal and tumor tissues. (**A**) Comparison of expression levels of the seven core prognostic lncRNAs between normal versus unpaired tumor tissues. (**B**) Comparison of expression levels of the seven core prognostic lncRNAs between normal versus paired tumor tissues. (**C**) Validation of the expression of the seven core prognostic lncRNAs in normal and tumor tissues using qRT-PCR. Statistical significance symbols: ns, $p \geq 0.05$; $^{*}p < 0.05$; $^{**}p < 0.01$; $^{***}p < 0.001$.

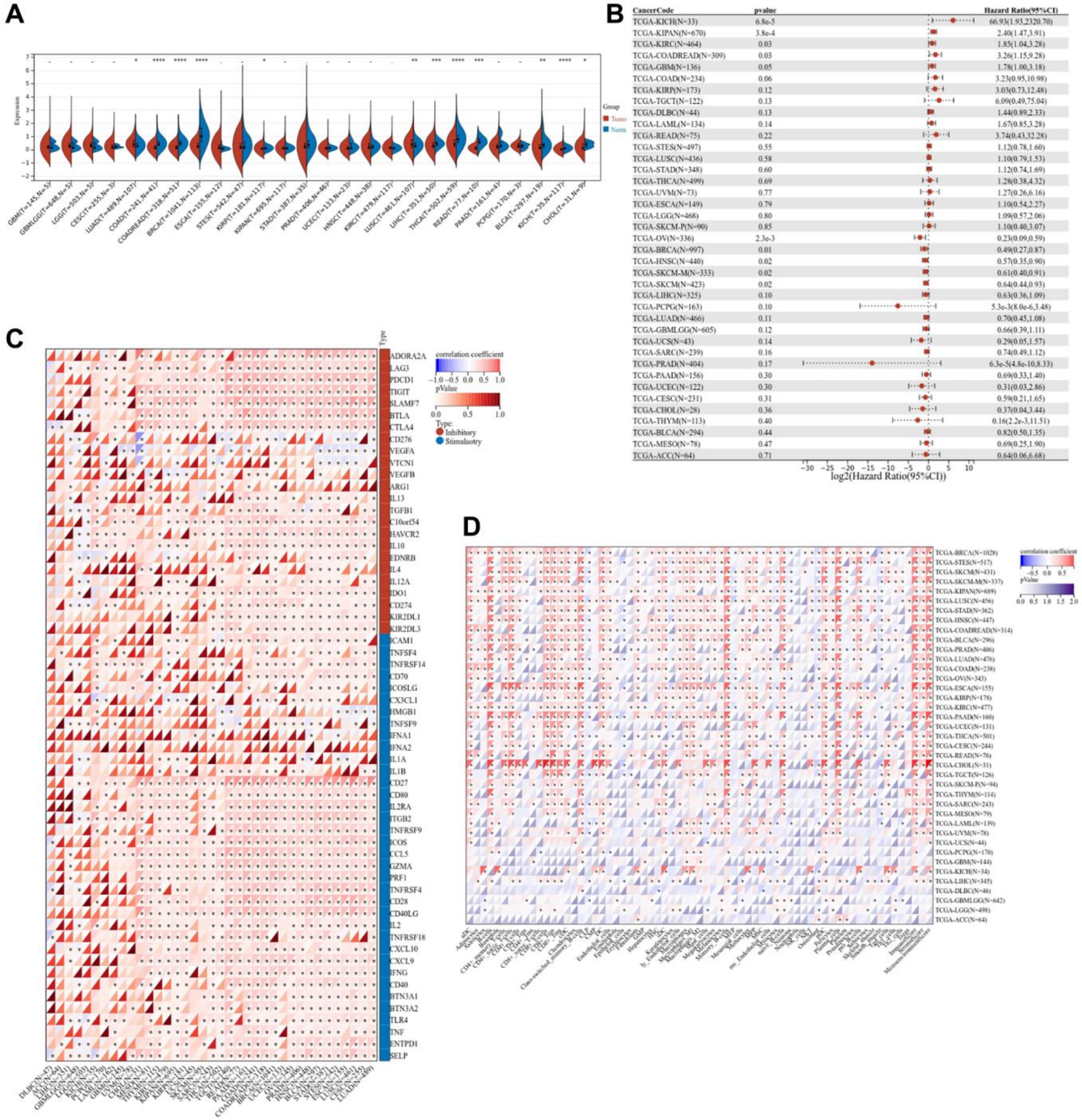

Supplementary Figure 6. Pan-cancer analysis of *AL133467.1*. (**A**) Expression levels of *AL133467.1* across various cancer types. (**B**) Univariate Cox analysis results showing the prognostic significance of *AL133467.1* in pan-cancer. (**C**) Correlation analysis of *AL133467.1* with immune regulation-related genes in pan-cancer. (**D**) Association between immune cell infiltration and *AL133467.1* expression in pan-cancer. Statistical significance symbols: ns, $p \geq 0.05$; $^{*}p < 0.05$; $^{**}p < 0.01$; $^{***}p < 0.001$; $^{****}p < 0.0001$.
