# Supplementary material for: Deciphering the prognostic significance of anoikis-related lncRNAs in invasive breast cancer: from comprehensive bioinformatics analysis to functional experimental validation
Source: Aging (Albany NY). 2024 Jan 5;16(1):402–30. doi: 10.18632/aging.205376 (PMC10817393; doi:10.18632/aging.205376)
Supplement: Supplementary Table 1 [file aging-16-205376-s002.pdf]

SUPPLEMENTARY TABLE

Supplementary Table 1. Primer sequences.

Genes	Forward primer	Reverse primer
C6orf99	CCACTCGAAGCCGGTGTCTG	AAGAGCAGATGGACAGCACGAC
LINC01614	GTGCCCTCACATGCCTCCAAG	GAAGACATCCTCAGCCCACCAC
LINC02613	CTGCGTGCCAAACTTGCTGAC	CCTGCCCTGGAAGTGCTTCG
AC055854.1	GGGAGAGTGGGGAGCAAACAG	AGGCAGAGGAGAGGCAGAAGG
AL133467.1	CTCCCCACCAGCAGAAACATCC	GCACAGGCACAGAGGCAGATAC
AC004585.1	TCTCTGGGACTGACCTGACTGC	CCCGCCCTGGTGCTCATTG
MAPT.IT1	TGGCTTGGCTCTTGGGTTCAC	AAAGTCACGCCCTTCCAGCAG
